# A Metagenomic Time-Series Approach to Assess the Ecological Stability of Microbial Mats in a Seasonally Fluctuating Environment

**DOI:** 10.1007/s00248-023-02231-9

**Published:** 2023-07-02

**Authors:** David Madrigal-Trejo, Jazmín Sánchez-Pérez, Laura Espinosa-Asuar, Jorge A. Valdivia-Anistro, Luis E. Eguiarte, Valeria Souza

**Affiliations:** 1 Departamento de Ecología Evolutiva, Instituto de Ecología, Universidad Nacional AutÓnoma de México, Mexico City, Mexico; 2 Facultad de Estudios Superiores Zaragoza, Universidad Nacional Autónoma de México, Ciudad de México, México; 3 Centro de Estudios del Cuaternario de Fuego-Patagonia y Antártica (CEQUA), Punta Arenas, Chile

**Keywords:** Extreme environments, Taxonomic replacement, Ecological resilience, Environmental perturbations, Rare biosphere

## Abstract

Microbial mats are complex ecological assemblages that have been present in the rock record since the Precambrian and can still be found in extant marginalized environments. These structures are considered highly stable ecosystems. In this study, we evaluate the ecological stability of dome-forming microbial mats in a modern, water-level fluctuating, hypersaline pond located in the Cuatro Ciénegas Basin, Mexico. We conducted metagenomic sampling of the site from 2016 to 2019 and detected 2250 genera of Bacteria and Archaea, with only <20 belonging to the abundant taxa (>1%). The microbial community was dominated by Proteobacteria, Euryarchaeota, Bacteroidetes, Firmicutes, and Cyanobacteria, and was compositionally sensitive to disturbances, leading to high taxonomic replacement even at the phylum level, with a significant increase in Archaea from $\sim$1-4% to $\sim$33% throughout the 2016-2019 study period. Although a core community represented most of the microbial community (>75%), relative abundances shifted significantly between samples, as demonstrated by changes in the abundance of *Coleofasciculus* from 10.2% in 2017 to 0.05% in 2019. Although functional differences between seasons were subtle, co-occurrence networks suggest differential ecological interactions between the seasons, with the addition of a new module during the rainy season and the potential shift in hub taxa. Functional composition was slightly more similar between samples, but basic processes such as carbohydrate, amino acid, and nucleic acid metabolisms were widely distributed among samples. Major carbon fixation processes included sulfur oxidation, nitrogen fixation, and photosynthesis (both oxygenic and anoxygenic), as well as the Wood-Ljundgahl and Calvin cycles.

**Supplementary Information:**

The online version contains supplementary material available at 10.1007/s00248-023-02231-9.

## Introduction

There is a wide consensus that life emerged and diversified relatively early in Earth’s history, as suggested by geochemical signatures, putative microfossils and biosedimentary structures from the early Archean [1]. Particularly, phototrophic non-lithifying microbial mats and stromatolites have been extensively documented in the Archean rock record, as shown in the fossil evidence from the Dresser formation (3.48 Ga) [2], the Buck Reef Chert (3.42 Ga) [3, 4], and the Moodies group (3.22 Ga) [5–7]. These microbial structures are also found in marginalized modern environments; they occur as benthic, stratified, and self-sustaining biological communities of thousands of phylogenetically diverse microorganisms embedded in a matrix of extracellular polymeric substances (EPS) [8, 9]. Given the morphological, chemical, and sedimentological similarities between modern and fossil microbial mats, it is straightforward to infer that these biological structures have been thriving on Earth for more than $\sim$3.5 Ga. As such, modern microbial mats are often considered analogs to benthic Precambrian communities in shallow-water environments, and therefore of paramount importance to better interpret the paleobiological record [10, 11].

The undeniable success of microbial mats can only be understood in terms of ecological stability; namely, the community response to disturbances, which can be dissected into the degree to which a community is insensitive to perturbations (ecological resistance) and the rate at which a community restores to the pre-disturbed state (ecological resilience) [12, 13]. Environmental disturbances can be classified into pulses and presses if the perturbation is a discrete, short-term event, or a continuous, long-term transition, respectively [12, 14]. Microbial community stability is a topic of interest for a wide array of systems and disturbances, such as dry-rewetting events [15], differences in water level [16], temperature variations [17, 18], chemical stress [19], shifting redox patterns [20], and changes in salinity [21]. Previous studies have shown that microbial communities commonly display high functional redundancy owing to the spatial coexistence of taxonomically distinct microorganisms, most of which belong to the rare biosphere [22, 23], while perturbations commonly have a negative effect on the species richness along with changes in the ecological interactions [24–27]. Despite recent advances, the impact of disturbances on the microbial dynamics of lithifying and non-lithifying microbial mats remains unclear [28–32]. A better understanding of these dynamics would provide insights into the ecological stability of these structures since the Precambrian.

**Fig. 1:**
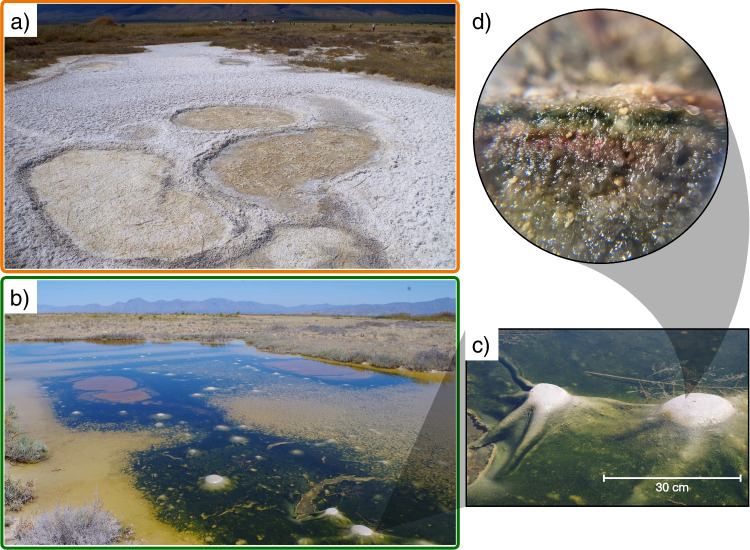
The Archean Domes microbial system. The pond ($\sim$50x25 m) displays different features during *a)* dry season (sampling of March 2019) and *b)* rainy season (sampling of March 2016). *c)* Detail of the dome-like structures of 10-15 cm in diameter. *d)* 10X magnification of the microbial mat; functional stratification and sediment grains can be appreciated at this scale. Photo credit for *a-c)*: David Jaramillo

The microbial mats in the Archean Domes system, in the Cuatro Ciénegas Basin (CCB), Mexico, endure extreme conditions. These conditions include extended droughts lasting approximately nine months each year, which could be classified as a press disturbance, as well as variations in salinity, pH, burial by precipitating salts, and shifts of trophic states. Additionally, daily temperature shifts amidst the desert and burial by sediments during heavy rainfall could be considered pulse perturbations. Thus, the Archean Domes system is best regarded as a multi-perturbation system. To gain insight into the underlying ecological processes and mechanisms that allow these microbial communities to flourish despite the seasonal disturbances that characterize this system, we employed a metagenomic approach to assess ecological stability and community dynamics over a four-year sampling period. We hypothesize that ecologically stable microbial mats would demonstrate no significant changes in community composition and functional profiles, whereas mats with high turnover rates would indicate limited resistance and resilience, potentially reducing the lifetime of the system. Our study aims to address several questions, including whether we can detect any differential responses between seasons, what is the contribution of the core community and rare biosphere to this system, and if we are able to observe any long-term genetic drift as a consequence of extreme environmental perturbations.

## Materials and Methods

### Study site and Sample Collection

In this work, we investigate the microbial system known as the Archean Domes, situated in the CCB, Mexico (Fig. 1). The Archean Domes (26$^{\circ }$49’41.7”N, 102$^{\circ }$01’28.7”W) is a seasonal, water-fluctuating pond in Rancho Pozas Azules from Pronatura Noreste located at the eastern side of the Cuatro Ciénegas Basin, Coahuila, México (Site overview in Fig. S1: Supplementary material). This site was discovered in 2016, and was firstly described by [33] [unpublished], [34], and [35]. During the rainy season, mostly during the months of August to September, the pond fills with water up to $\sim$20 cm. Green mats emerge over the sediment surface (i.e., epibenthic microbial mats), building dome-like sedimentary structures up to 10-20 cm in diameter (Fig. 1b,c). Although unusual in morphology and size, similar structures have been previously reported in several hypersaline, intertidal and supratidal environments [36–38]. Descriptions of gas domes and other Microbially Induced Sedimentary Structures (MISS) from these non-lithifying microbial mats can be found in Fig. S2: Supplementary material. From November to July, water evaporates and salt precipitation covers the pond completely, burying the microbial mats (Fig. 1a). Salinity is variable between the two seasonal states, transitioning from 52.5‰ (as measured in the rainy season of 2016) when filled with water to salt saturation during the dry season. From a recent sampling in September 2021, we observed that green mats and gas filled structures start to quickly develop after a day of rainfall (Fig S3: Supplementary material). Inside the dome-like structures of the mats, variable concentrations of methane (2.6$-$19.6 $\mu$g/L on the dry season of 2016, 102-402 $\mu$g/L on the dry season of 2017) and carbon dioxide (1.08$-$1.40 mg/L on the dry season of 2017) were measured (Table S1: Supplementary material). Chemical analyses for organic and inorganic nitrogen (ON/IN), phosphorus (OP/IP), and organic carbon (OC) were determined for the dry and rainy seasons of 2019 (Table S2: Supplementary material); on average, molar ratios for the 2019 dry season are 2.37 for OC:ON, 152.72 for OC:OP, and 77.61 for ON:OP, while the 2019 wet season shows 1.56 for OC:ON, 16.98 for OC:OP, and 9.96 for ON:OP. During dry season, pH is $\sim$5$-$8.6, while on rainy season the pH rises to $\sim$8.2$-$9.5 with the dissolution of salts. Water/mat temperature is $\sim$31.5$-$34.5 when sampled (always during daylight). Sediment grain size at this site is dominated by sand (66.1%$-$68.3%) followed by silt (23.4%$-$24.0%) and clay (8.5%$-$9.9%), which correspond to a sandy loam soil texture (Table S2: Supplementary material). For details on equipment and physicochemical methods, confer the following section.

We collected six samples of mats and associated sediment across a four-year period (See photo gallery for each sampling in Fig S3: Supplementary material). During this time span, we got to collect three samples of each seasonal state: dry and rainy season. The mats from the dry season are from the sampling of April 2016, February 2017 and March 2019 (hereinafter denoted as Dry-2016, Dry-2017 and Dry-2019, respectively); mats from the rainy season are from the sampling of October 2016, October 2018 and September 2019 (hereinafter denoted as Wet-2016, Wet-2018 and Wet-2019, respectively). Rainy season samples come directly from developing domes, whereas dry season samples derive from regions where mats were visible to the naked eye. As the rainy season is heavily contingent on the cyclone dynamics of the Gulf of Mexico, samples were taken at different times, $\sim$1-2 weeks after a heavy rainfall to ensure a high level of water in the pond. To prevent contamination, samples were collected with gloves, sterile forceps and sterile conical tubes (50 mL). Samples were stored at 4 $^{\circ }$C and subsequently frozen in liquid nitrogen for preservation, prior to DNA extraction. Samples were collected under the collection permit SGPA/DGVS/03188/20 issued by Subsecretaría de Gestión para la Protección Ambiental, Dirección General de Vida Silvestre (https://www.gob.mx/semarnat). Weather parameters during sampling were taken from the National Meteorological Service, CONAGUA (https://smn.conagua.gob.mx/es/), at the EMA station No. 15DBB372 in Cuatro Ciénegas (27$^{\circ }$0’7.2”N, 102$^{\circ }$4’22.7”W). Weather data is provided in Fig. S4 and Table S3: Supplementary material.

### Measurement of Physicochemical Parameters

Water salinity, temperature, and pH were measured *in situ* using a Hydrolab MS5 Water multiparameter sonde (OTT Hydromet GmbH, Germany). Prior to chemical analyses, samples were air dried, sediments were sieved through a 2.0 mm sieve and subsequently ground in an agate mortar. The pH of the sediment was determined using a Thermo Scientific (Waltham, Massachusetts) H03062 digital pH meter [39]. Total organic carbon (TOC) and organic matter (OM) content were quantified using the Walkley-Black method [40]. The OM content was calculated with the conversion factor 1.298 (1/0.77) used for Mexican soils. Total nitrogen (TN) was determined with the micro-Kjeldahl method (salicylic acid-thiosulfate modification, [41]), while inorganic nitrogen (IN) was determined by direct-distillation of the sediment extract [42]. Organic nitrogen (ON) was estimated as TN minus IN. Total phosphorus (TP) and inorganic phosphorus (IP) fractions were extracted with bicarbonate [43]. Both P fractions were reduced with ascorbic acid and quantified by the molybdate colorimetric method [44]. The organic phosphorus (OP) fraction was estimated by the difference between TP and IP. Finally, the sediment texture was determined by the Bouyoucos hydrometer method [45].

Dissolved CH$_4$ and CO$_2$ concentration inside the domes were determined with a discrete headspace. equilibration technique. Briefly, triplicate water samples were collected with 60 mL plastic syringes, ensuring the absence of air bubble. Then, 20 mL of water was gently evacuated and substituted with CH$_4$- and CO$_2$-free nitrogen (99.999 % N$_2$, Praxair, Mexico). The syringe content was vigorously shaken for equilibration, the liquid volume was evacuated, and 5 mL of subsample of the gas content of the syringe was injected into a continuous flow of nitrogen connected to an ultraportable greenhouse gas analyzer (UGGA, Los Gatos Research, CA, USA). The presence of CH$_4$ and CO$_2$ in the gas sample was detected as a peak response, that was integrated, after proper calibration with standard CH$_4$ /CO$_2$ samples. Lastly, dissolved CH$_4$ and CO$_2$ concentration were derived from the gas concentration using the Henry’s solubility constant of both gases [46].

### DNA Purification and Sequencing

From each sample, only the mat layer ($\sim$1 cm) was taken for DNA extraction. As the samples of the dry seasons contain a thick layer of salt, this layer had to be separated with a sterile scalpel to facilitate the extraction. We perform the extraction of total DNA from the six samples as reported in [47]. Purified DNA was sent to CINVESTAV-LANGEBIO (http://langebio.cinvestav.mx/labsergen/) for shotgun metagenomic sequencing. DNA libraries for Illumina paired-end sequencing were prepared using Ilumina TruSeq DNA Nano; no amplification steps were performed. Library quality control was performed with Aligent Bioanalyzer High Sensitivity DNA Analysis Chip. DNA from all samples was sequenced with Illumina MiSeq, 2 x 300 bp paired-end reads format. The total number of paired-end reads per metagenome range from 4.7 to 28.0 Gbp per library and orientation (forward and reverse). Number of raw reads and quality control metadata can be found in Table S4: Supplementary material.

### Quality Control, Assembly and Annotation of Metagenomes

We preprocessed the raw reads with Trimmomatic v0.38 [48] with a sliding window of 4 bp, a Phred quality score of 30, minimum length of 35, and an average mean quality of 28. For each metagenome, reads were assembled into contigs to facilitate gene prediction. Forward and reverse paired reads, and individual forward and reverse with no pair, were assembled using MEGAHIT v1.1.1 [49] with minimum contig length of 500, k-min of 27 and k-step of 10 as suggested for highly-diverse metagenomes [50, 51]. To control for sequencing depth bias, we used the minimum number of reads (1,288,875 reads) to sample the metagenomic datasets at random to normalize coverage for comparisons. Unassembled reads were collected with BBtools [52] and SAMtools v1.12 [53]. For assembled contigs, gene prediction and subsequent taxonomic annotation was done with CAT v5.2 [54]. CAT is a robust taxonomic annotator that integrates known software programs such as gene predictor Prodigal [55] and gene annotator DIAMOND [56] against the NCBI non-redundant database [57] to give a deep gene taxonomic annotation. Since taxonomic annotation with CAT revolves against all kinds of predicted genes, we also used six ribosomal protein families (PF00177, PF00298, PF00573, PF00237, PF00163 and PF00318) to validate CAT results. We downloaded ribosomal genes’ seeds from Pfam database [58]. HMM profiles were built with HMMER v3.3 [59], and hmmsearch was performed against all metagenomes (e-value 10$^{-6}$). Ribosomal genes were annotated with DIAMOND, coupled with the NCBI non-redundant database. Additional information regarding quality control, metagenome assembly, processing of not assembled reads with MEGAHIT, and taxonomic annotation (CAT and ribosomal) can be found in Table S4-S8: Supplementary material. Functional profiling for each sample was performed with SUPER-FOCUS [60] against the NCBI non-redundant database (https://www.ncbi.nlm.nih.gov/). Additionally, we selected resistance genes based on GO classification [61, 62] and downloaded the amino acid sequences from UniProt database. Selected GO terms are shown in Table S9: Supplementary material. Resistance query sequences were aligned with BLAST [63] against all metagenomes. Finally, we selected key energy metabolisms as in [64]. Protein families involved in each metabolic pathway were initially searched in UniProt [65] and KEGG [66] databases, and subsequently downloaded from Pfam

### Normalization, Statistical Analyses and Data Visualization

We used R programming language [67] to run each statistical analysis, to normalize data, and to generate figures. We list the libraries used as follows: ggplot2 v3.3.5 [68] for sockplots and boxplots, edgeR v3.34.1 for data normalization [69], RAM v1.2.1.7 [70] for PCoA, PCA and CCA analyses, vegan v2.5-7 [71] for rarefaction curves and alpha-diversity metrics, UpSetR v1.4.0 [72] for upset plots, DESeq2 v1.32.0 [73] and EnhancedVolcano v1.10.0 [74] for differential expression analysis, patchwork v1.1.1 [75] and fmsb v0.7.1 [76] for radar charts, streamgraph v0.9.0 [77] for streamgraphs, easyalluvial v0.3.0 [78] and parcats v0.0.3 [79] for alluvial plots, NetCoMi v1.0.2 for network analyses [80], and umap v0.2.7.0 and dbscan v1.1-8 for clustering. Libraries BBmisc v1.11 [81], dplyr v1.07 [82], tidyr v1.1.4 [83] were used for data manipulation. Gene abundances were normalized with the Relative Log Expression (RLE) method. PCoA and NMDS analyses for taxonomic groups were calculated with a Bray-Curtis measure. Co-occurrence NetCoMi networks were grouped by seasons in order to minimize environmental indirect edges, a relevant effect reported elsewhere [84]. The networks were built using SparCC measure, Bayesian-multiplicative replacement for zero handling and association threshold of 0.5. Phylum-level networks were built with the top 120 phyla, while genus-level networks were built with all the 250 core genera. Groups of taxa and functions clustered with UMAP/HDBSCAN and k-means, respectively, are provided as supplementary.csv files.

## Results and Discussion

### Functional Inference and Changes Through the Seasons

**Fig. 2:**
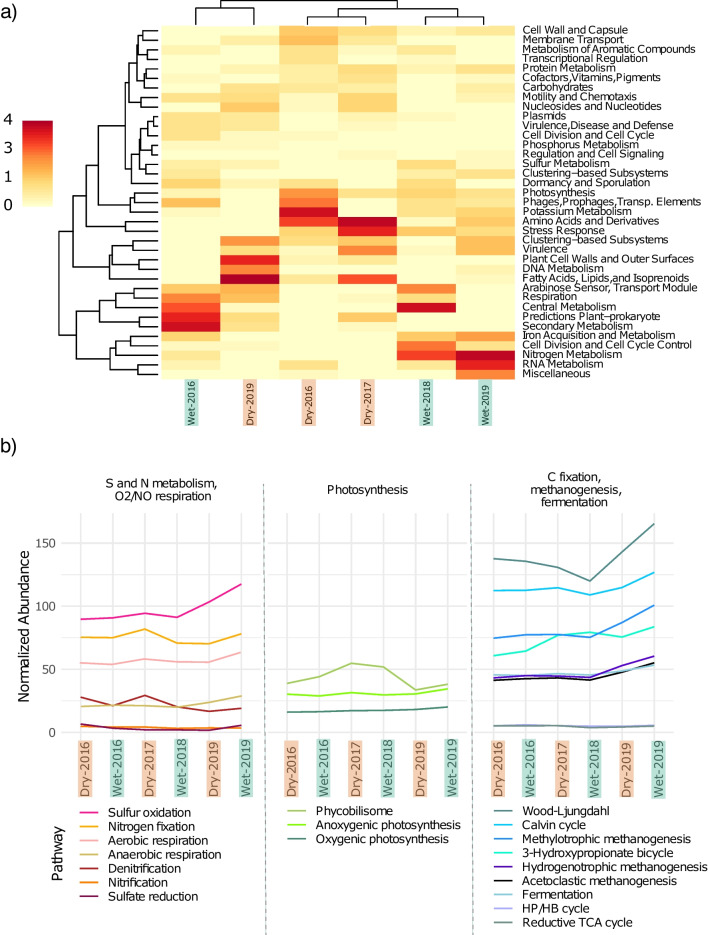
Potential functional profile based on metagenomic inference. *a)* Heatmap of SUPER-FOCUS major functions, with color intensity reflecting differences in normalized abundance between samples. *b)* Normalized abundance of selected pathways based on pathway-specific Pfam protein groups. HP/HB=3-hydroxypropionate/4-hydroxybutyrate, TCA=tricarboxylic acid

Coding sequences were functionally classified. As expected, basic functions shared between all living beings are widely distributed among all samples, such as carbohydrate (14.5%), amino acid (11.9%), protein (8.9%), DNA (5.9%), RNA (5.0%), and fatty acids and lipids (3.1%) metabolisms; other processes regarding cofactor, vitamins, and pigments (10.7%), cell wall and capsule (4.2%), respiration (3.9%), and stress response (3.8%) are also among the top functions for all samples (Table S10: Supplementary material). The fact that stress response genes appear in a relative high abundance is plausibly an adaptation to a community that is subject to ceaseless environmental pressures [85, 86], such as those found in the Archean Domes system. Differences in function abundance for every major process according to SUPER-FOCUS classification are depicted in Fig. 2a. Overall, samples appear to be similar among them, despite some functions with differential distribution among the samples, such as amino acid, fatty acids and lipids, central, secondary, nitrogen, potassium, and RNA metabolisms.

We inspect in detail the functional role of stress response genes present at the Archean Domes. Using the GO classification, we identify resistance genes related to pH (both, alkaline and acidic genes as well as not specified, general pH control genes) salt, dormancy, and endosporulation conditions. Alkaline and salt resistance genes were the most abundant, with a mean proportion of 56.5% and 31.5%, respectively (Fig. S5: Supplementary material). This behavior is expected, since salt and pH fluctuate considerably between seasons, and might exert a selection pressure on the organisms thriving on this site.

Pfam protein groups were used to infer energy metabolisms and nutrient cycling within the mat samples (Fig. 2b). Based on normalized abundance, Wood-Ljungdahl pathway rules carbon metabolism among the mat, followed by the Calvin cycle. These results are consistent with other microbial mats previously described, where Wood-Ljungdahl dominance is regarded as a result of energy limitation, since this mechanism of carbon fixation is inefficient compared to other pathways [64, 87, 88]. Anoxygenic photosynthesis genes dominate over those specific to oxygenic photosynthesis, while sulfur oxidation and nitrogen fixation are potentially the main processes for sulfur and nitrogen metabolisms. Dissimilatory sulfate reduction is portrayed as a process with low gene abundances in the Archean Domes, despite the highly abundant sulfate reducing bacteria previously described; as such, metabolism inference based on gene abundances should be taken cautiously. Further reconstruction of full pathways would provide more accuracy in the relative abundances.

**Fig. 3:**
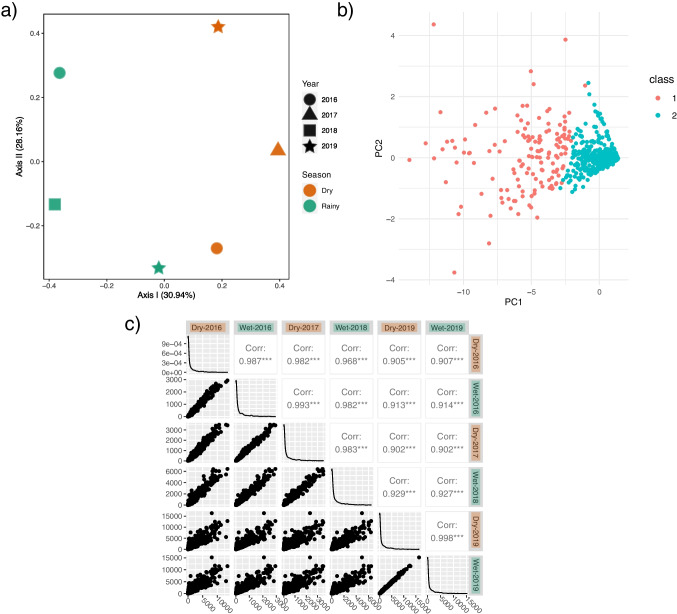
Functional comparison between seasons and functional changes throughout the years. *a)* Dry and rainy season comparison based on top SUPER-FOCUS processes. Similar abundances can be inferred from both dry and rainy seasons (green dots lay mostly on top of the orange dots). *a)* PCoA of functions with Bray-Curtis measure, where samples are grouped, to some extent, by season. *b)* PCA analysis showing main groups of functions by k-means clustering. cluster 1 (red) shows high sparsity, and it is associated with the most abundant functions, while cluster 2 (blue) has functions with lower abundance. *c)* Correlations of function abundance between each sample, where each sample is more similar to the adjacent ones in time. X and Y axes show number of counts

Ecological resilience is not straightforward to assess in this system, as press disturbances are continuous and seasonal. Rather, inferences on ecological resilience could be evaluated under the assumption of many stable states in which a community may thrive. PCoA plots could be visualized as stability landscapes, where each snapshot of the community’s composition/function could be envisioned as a ball and where the different alternative stable states represent basins in the stability landscape. If resistance and resilience is high, a disturbance would not modify the current stable state of the community. On the contrary, if overall stability is low, and the disturbance powerful enough, the community will leave its current stable state to fall into an alternative stable state [for an in-depth review on the stability landscape, cf. 12, 89]. Looking at the PCoA plot with a Bray-Curtis measure for functions (Fig. 3a) we could see that seasonal “valleys” of alternative equilibria are formed, and that these equilibrium states are arranged into seasonal groups, although ordination is sparse. Further robust sampling will support the predictability of this clustering method.

We modify a differential expression analysis to adapt it to our metagenomic using the classification defined by SUPER-FOCUS. Although none of the metabolic subsystems had a significant difference between seasons (*p* > 0.5), there were some processes that had a higher or lower abundance as seen by their fold change (Log$_2$ Fold Change $>|2.5|$, Fig. S6: Supplementary material). In the dry season, there were three slightly more abundant functions: the pentose phosphate pathway of plants, the alpha-acetolactate operon, and the biotin biosynthesis. From the pentose phosphate pathway, we had the glucose 6 phosphate dehydrogenase, the key enzyme of the Oxidative Pentose Phosphate Pathway (OPPP), which is related to the response of short- or long-term exposure to drought stress in plants [90]. The alpha-acetolactate operon has been described as a component in the mixed acid fermentation, done by some bacteria such as *Bacillus subtilis*, to produce acetoin in the absence of nitrate [91]; this could be associated to a shortage of nutrients in the dry season. Lastly, biotin biosynthesis is an important process, since biotin is a key cofactor in the fatty acids and amino acid metabolisms, as well as in the replenishment of the tricarboxylic acid cycle [92] which could be a relevant resistance process at low water activity. For the rainy season, some functions with a higher fold change were: the acyl homoserine lactone (AHL) inducer, which is involved in primary quorum sensing signals by Gram-negative bacteria [93]; the phage carbon metabolism auxiliary metabolic genes (AMGs), which consist of phage strategies for resource management during host infection [94, 95]; some archaeal hydrogenases involved in carbon fixation [96]; prenylated indole alkaloids production from actinomycetes, which have multiple biological functions such as antifungal and antibacterial activity [97]; and lastly, chlorophyll degradation related genes. Some of these functions could be directly associated with the presence of the green, cyanobacterial built, layer seen in the rainy seasons, such as in the phage-cyanobacterial AMGs [94], the quorum sensing for biofilm formation [98], and the chlorophyll degradation.

Moreover, we were interested in how functions have changed over time and if there is a group of functions that leads global patterns in the community. According to k-means and hierarchical clustering, two main groups of functions were predicted. PCA analysis showed how these functions are projected, where high abundant functions are sparsely distributed in the plot, and most functions with low abundance functions were tightly clustered together (Fig. 3b). Each function’s class is provided as a supplementary.csv file. Comparing function abundance across samples suggest that functions are more similar between adjacent samples (Fig. 3c), despite the high similarity in function abundance for each sample. In consequence, the correlation cloud appears to be scattering when samples are more distant in time. For instance, sample Dry-2016 showed higher correlation with sample Wet-2016 than with the last sample from 2019 (Wet-2019). While major functions appear to be constant over time, this result suggests that overall functions are changing in abundance, perhaps slightly, between samples, and that cumulative changes in abundance for the final sample differ drastically from the initial function state. Further sampling may reinforce this hypothesis.

Since functions are mostly preserved (in abundance) through the seasons, the microbial community might harbor a high degree of functionally redundant taxa [24]. This grants the community a robust capability to withstand taxonomic replacement, a phenomenon detected at the Archean Domes system which is the outcome of a non-resistant community in terms of phylogenetic composition (see the following section).

### Taxonomical Characterization, Compositional Dynamics, and Seasonal Comparison

We built rarefaction curves to evaluate diversity coverage for all samples. For genera richness, each sample reaches saturation and comparisons between them is suitable (Fig. S7: Supplementary material). Open read-frames were predicted for reads, and further annotated for taxonomic classification with CAT and ribosomal protein families. With CAT, we detected 162 phyla, 2250 genera (across all samples), and more than 8,000 phylotypes per sample (Table S7: Supplementary material). Nevertheless, only 30-58% of the total predicted genes for each sample were classified, suggesting a considerable amount of potential novel taxonomic groups, which comprise the so called microbial dark matter; these potentially uncultured organisms are by no means irrelevant, as they have shown to be of importance in other hypersaline microbial mats [99]. From the 2250 total genera found in the system, only between 16-19 for each sample belong to the abundant genera, that is, with an abundance >1%. In contrast, between 426-619 genera have abundances <0.1%, and belong to the so called rare biosphere. Rare taxa account for the 11.2$-$18.9% of the whole community, whereas abundant taxa comprises the 43.3$-$67.6% (Table S11 and Fig. S8: Supplementary material). Therefore, although taxa that are abundant only consists of a few genera, these taxa often build most of the microbial community biomass. Moderately abundant taxa (>0.1% and <1%) sits between the abundant and rare, with a relative abundance of 19.6$-$37.7% in the samples studied.

**Fig. 4:**
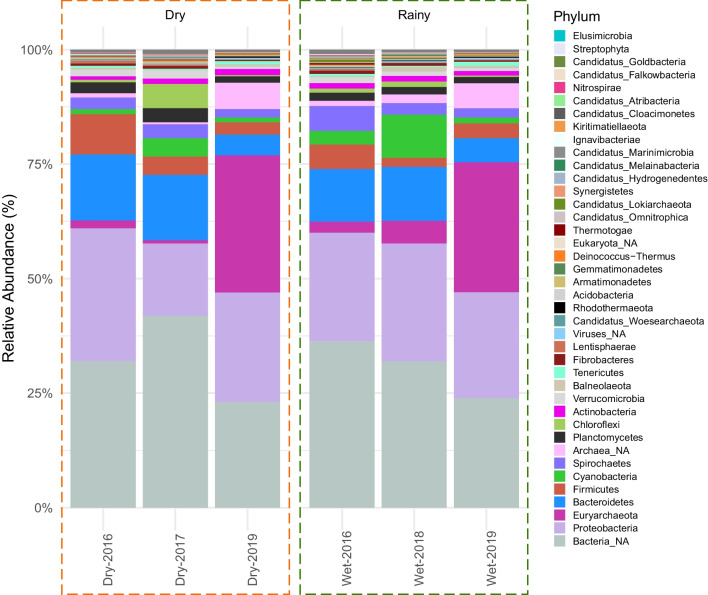
Taxonomic profile of the Archean Domes based on CAT annotation. Only the top abundant phyla are displayed. Not annotated phyla were grouped into NA category for each superkingdom, accordingly. Sequences not annotated at the superkingdom level are not shown

Regarding the taxonomic composition, overall, mean abundances per domain show consistent results between CAT and ribosomal gene annotation. For CAT taxonomic assignment we got mean abundances of: 85.24% for Bacteria, 14.43% for Archaea, and 0.3% for Eukaryota (Table S7: Supplementary material); whereas ribosomal gene annotation showed: 86.56% for Bacteria, 13.35% for Archaea, and 0.08% for Eukaryota (Table S8: Supplementary material). At the phylum level, samples consistently displayed Proteobacteria (23.51%), Euryarchaeota (11.42%), Bacteroidetes (10.26%), Firmicutes (4.35%), Cyanobacteria (3.30%), Spirochaetes (2.84%), Planctomycetes (1.99%) and Chloroflexi (1.42%) as the most abundant phyla (Fig. 4). The taxonomic annotation with ribosomal genes is also consistent with the phyla relative abundances of CAT annotation (taxonomic profile based on ribosomal proteins is shown in Fig. S9: Supplementary material).

At the genus level, we find *Coleofasciculus* as the most abundant Cyanobacteria between all samples, which is widely known as a key mat-forming genus in sandy environments [9, 11, 100, 101]. Other cyanobacterial genera such as *Leptolyngbya*, *Halothece*, and *Phormidium* are also abundant between samples, and have also been previously reported in microbial mats [100, 102, 103]. Anaerobic, halophilic, sulfate-reducing members of the Deltaproteobacteria such as *Desulfonatronovibrio*, *Desulfonatronospira*, and *Desulfovermiculus* [104] also appear in abundance in the Archean Domes samples. Other relevant and abundant taxonomic genera present in the samples include *Halorubrum* (Euryarchaeota), *Halanaerobium* (Firmicutes), *Spirocheta* (Spirochetes), *Chitinispirillum* (Fibrobacteres), and *Tangfeifania* (Bacteroidetes).

**Fig. 5:**
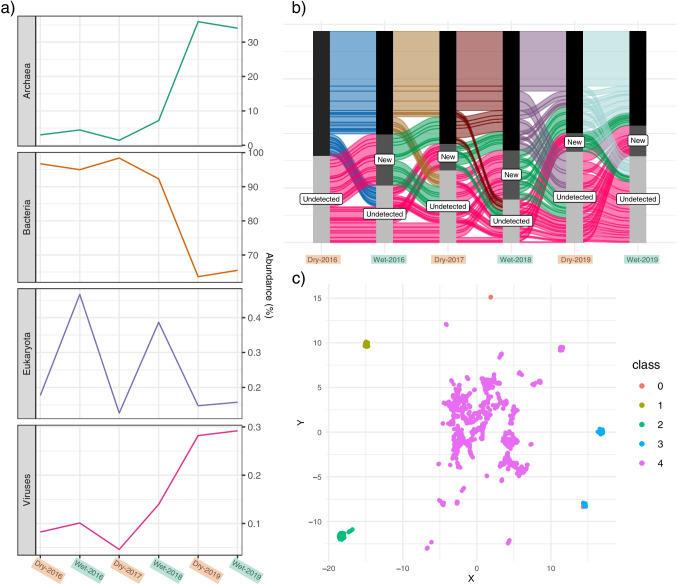
Taxonomic replacement and community dynamics throughout the years. *a)* Changes in superkingdom abundances from 2016 to 2019. Notice differences in scales. *b)* Community dynamics at the genus level. Flows show genera that remain, appeared or disappeared from the system through the samples. Genera that are not detected within a given sample are grouped as undetected. Undetected genera that appeared in the subsequent sample are grouped as new genera, whereas they remain in the undetected group otherwise. Genera detected in one sample can also become undetected genera in the one that follows. *c)* UMAP dimension reduction and HDBSCAN clustering technique applied on the differences in genus abundance for each sample. 5 clusters were generated (0-5)

Figure 5a show the community composition changes through the years. One of the most noticeable changes through the years was a rise of Archaea (from $\sim$1-4% to $\sim$33%) in the samples of 2019 (Tables S5 and S6: Supplementary material). Consequently, Bacteria reduced their abundance up to $\sim$65%, a third less from previous years. Viruses followed the same tendency as the Archaea during 2019, in a subtle rise of abundance ($\sim$0.08$-$0.2% to $\sim$0.4%), while Eukaryota had an apparent seasonal pattern in the first two years (2016–2018), continuing with a steady state in 2019. Since the increased abundance of Archaea was considerable, the compositional dynamics between 2016-2018 is not readily noticeable. Considering only the abundance shifts between 2016-2018, all domains presented a plausible seasonal pattern: archaea, eukaryotes and viruses rose proportionally in the rainy season compared to the dry one. To explore which organisms may drive these seasonal patterns, we examined phyla changes in abundance through time. Interactive streamgraphs for phyla shifts are provided as.html supplementary files. Phyla with prominent shifts were Spirochaetes, Proteobacteria, Cyanobacteria, Cloroflexi, Bacteroidetes, and the Euryarchaeota. Euryarchaeota became one of the main groups in the communities of 2019 (from $\sim$2% to $\sim$28%), and the phylum responsible for the overall increase in Archaea. In contrast, Cyanobacteria, Chloroflexi and Bacteroidetes showed a diminished abundance during that same year. Spirochaetes had increased in abundance in October 2016 to end in a constant frequency in the following samples. From this initial domain and phyla description, we could infer that the community’s composition is heavily affected by each seasonal-and overall, temporal-shift(s), pointing towards a sensitive, non-resistant, microbial community [105].

At the genus level, the taxonomic replacement is even more noticeable (Fig. 5b). For each sample, we could observe two main phenomena: i) some genera are present in every sample (core genera), while ii) most genera are new additions or become undetected between each sample. As a matter of fact, taxonomic replacement becomes increasingly complex with each new sample, which reflects how the community has changed since the first sampling in April 2016. We were interested to evaluate which taxa are key in driving the community to new compositional states, based on the differential abundances between samples. Coupling UMAP (Uniform Manifold Approximation and Projection), a nonlinear dimensionality reduction method, with HDBSCAN (Hierarchical Density-Based Spatial Clustering of Applications with Noise), a clustering algorithm, we find groups that might be leading the community dynamics (Fig. 5c). First, the main cluster contains most of the genera, with the inclusion of all the abundant taxa (1734 genera in class 4). In contrast, four small groups with fewer genera in each one (22, 116, 200, and 182 genera in classes 0,1,2, and 3, respectively. Group composition is supplied as a supplementary.csv file); these groups are made up entirely of genera belonging to the rare biosphere, and shifts in their abundance seem to be major ecological drivers in the system. This result could further support the relevance of the rare biosphere in biogeochemical processes and microbial community assembly, particularly, under fluctuating conditions as those met in the Archean Domes system [for reviews on rare biosphere, see 22, 106].

On account of the morphological changes of the pond in response to environmental perturbations, we conduct statistical analyses to evaluate if samples have higher resemblance to those collected in the same seasonal state. Alpha diversity indexes were calculated for each sample (Table S12 and Fig. S10: Supplementary material), and no statistical significance was found between seasonal states (Wilcoxon Rank Sum test: Chao1 *p*=0.4, Shannon *p*=1, Inverse Simpson *p*=0.8); nonetheless, the Archean Domes have a high alpha diversity for both seasonal states as seen in Chao (143-271), Shannon (2.5$-$3.1) and inverse Simpson (4.6$-$8.3) indexes, and overall, the microbial system during the 2016-2019 dry seasons is less diverse. This fact is remarkable, as ecological resistance has been classically associated with the species/genera richness, where an increase or decrease in biodiversity could point to a decrease in compositional stability [107].

Principal coordinates analysis (PCoA) and non-metric multidimensional scaling (NMDS) at the genus, order, and phylum level showed no seasonal aggregation of samples (Fig. S11: Supplementary material); rather, three temporally-related groups seem to have formed: one with both 2016 samples, a second group with the 2017-2018 samples, and a last one more closely arranged which comprises the samples from 2019. This last cluster is expected, as taxonomic profiles from the dry and rainy seasons of 2019 showed noticeable similar compositions (Fig. 4) as noted previously (i.e., an increase in the Archaea relative abundances). Interpreting the resulting PCoA plot for taxonomic composition as a stability landscape (as mentioned above), it seems that the community transitioned towards different compositional states from 2016 until 2018, but in the 2019 samples the community remained in the same compositional equilibrium state. Aditionally, canonical correspondence analyses (CCA) were performed at the genus and phylum level along with the environmental variables provided by the EMA meteorological station (Fig. S12: Supplementary material). From these analyses, apparently, 2017-2018 samples were driven by precipitation, whereas 2019 samples were driven by wind speed and humidity. These associations should be regarded with care due to the low sample number.

Co-occurrence networks were built for each seasonal state to inspect general properties at the phylum level (Table S13 and Fig. S13: Supplementary material). Both seasons show mainly two clusters, which might be interpreted as groups of highly interacting organisms or functional guilds with niche overlapping to some degree [84]. During the rainy season, several phyla from both groups transition to build a third cluster; hence, it is possible that these fluctuating environmental conditions directly influence differential interactions between phyla or niche overlaps, plausible consequences of phenotypic plasticity [108–110]. The phylum-level networks showed a positive edge percentage of 49.02% and 48.77% during the dry and rainy seasons, respectively; this network metric has been used to evaluate resilience and resistance in microbial co-occurrence networks: high positive/negative ratios, such as those found in these networks, have been interpreted to increase the community stability by avoiding feedback loops in taxa with overlapping niches [26]. High modularity has also been considered as a measure of community stability, diminishing the propagation of perturbations through the network [26, 111]. Modules in these networks might reliably represent functional guilds or niche overlapping [26, 84], and the Archean Domes microbial mats seem to change in modularity between the dry season (0.01) and rainy season (0.07) states. Lower modularity during the dry season might reflect the exposure and vulnerability of the system relative to when the mats are wet. We restrain to make inferences on specific biological interactions, as the small sample size might induce spurious correlations in the microbial networks.

### The Core Community

**Fig. 6:**
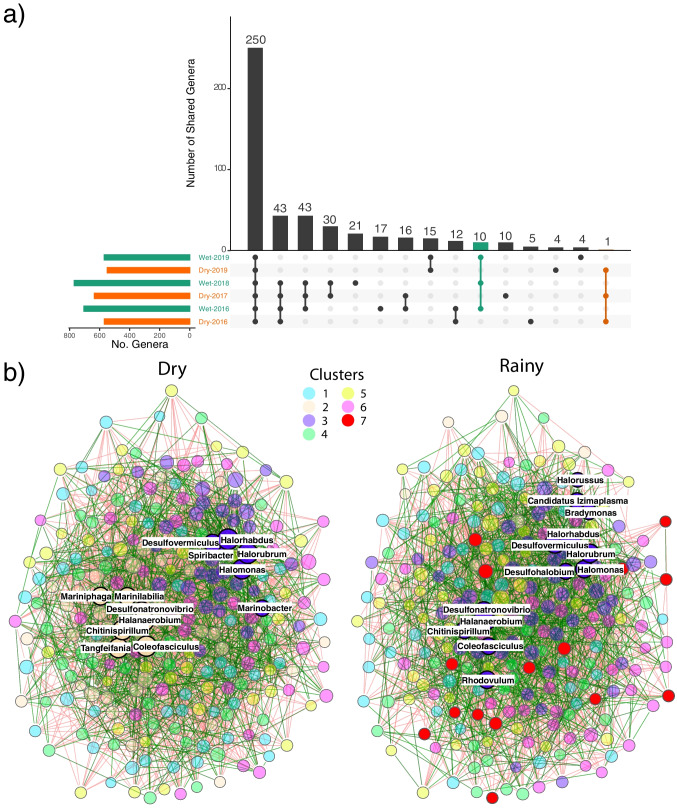
The core community at the Archean Domes microbial system. *a)* Upset plot showing the number of shared genera between different sample intersections. Global core showed 250 genera, while rainy core and dry core showed 10 and 1 genera, respectively. *b)* Co-occurrence networks for core genera during the dry and rainy seasons, where color indicates different clusters. Six clusters are shared between dry and rainy seasons. Note the addition of a red cluster during the rainy season. Green and red edges represent positive and negative relationships, respectively. Hub taxa are shown with labels

Taxonomic composition and functions changed through time to some extent, as described in the previous sections. Still, there is a core community shared between all samples and seasons. The (global) core community consists of 250 genera out of the 967 total genera detected across the samples; just about $\sim$26% of the total diversity found in the Archean Domes (Fig. 6a). Conversely, the core community accounts for much of the genera relative abundance, ranging from 75.6%, 77.5% and 78.7% in samples Dry-2019, Wet-2019 and Wet-2016, respectively, to 80.8%, 81.4% and 92.9% in samples Dry-2017, Wet-2018 and Dry-2016, accordingly (Fig. S14: Supplementary material). These genera can be portrayed as microbes with high physiological plasticity, able to cope with both environmental conditions (dry and rainy seasonal states) [20]. Seasonal cores were identified, that is, genera that only appeared in rainy or in dry season exclusively. Unlike the core community, seasonal cores were particularly small, with just 1 and 10 genera for dry and rainy seasons, accordingly (Fig. 6a). Every genus in the seasonal cores have a low abundance (<0.01%), and belong to the rare biosphere during each season. The organisms found only in rainy samples comprise several Alphaproteobacteria (*Croceicoccus*, *Shimia*, *Rhodoplanes*, and *Polymorphum*), Gammaproteobacteria (*Teredinibacter* and *Allochromatium*), Bacteroidetes (*Ohtaekwangia*), Cyanobacteria (*Geminocystis*), one Euryarchaeota (*Methanosalsum*) and a novel genus of Planctomycetes (Candidatus *Jettenia*) previously described in an anammox bioreactor [112]. Among these genera present only in rainy season, it is noticeable the presence of the phototrophs *Allochromatium* (purple sulfur bacteria), *Rhodoplanes* (photoheterotroph) and *Geminocystis* (oxygenic phototroph) [113, 114]. Recently, a *Croceicoccus* species has been found to be capable to produce AHL [115], which could be consistent with the slight increase in the AHL inducer genes during the rainy season. *Teredinibacter* have nitrogen fixation capabilities [116], while *Methanosalum* is a methylotrophic methanogen [117], which might aid in nutrient cycling during the rainy season. In contrast, the dry season core only contained the *Maledivibacter* genus, a member of the Clostridiales, Firmicutes. This genus produces hydrogen sulfide and ammonia under obligately halophilic conditions [118]. In fact, all the genera found in the seasonal cores are halophilic to some extent.

We further analyzed the taxonomical structure and functions of the global core community. Most of the 250 core genera belong to the Proteobacteria (102), Bacteroidetes (43), Firmicutes (28), Euryarchaeota (12), Actinobacteria (11), and Cyanobacteria (10) (Fig. S15: Supplementary material). Although these genera appear in every sample, their relative abundances fluctuate drastically between samples (Fig. S16: Supplementary material). For instance, *Coleofasciculus* transitioned from being one of the genera with the highest abundance (10.2%) in 2017 to belong to the rare biosphere during the dry and rainy season of 2019 (0.06% and 0.05%, respectively). This change in abundance could be responsible for the reduced dome size and abundance in later samplings (cf. Figure S2: Supplementary material), as EPS production has significant relevance in containing gases, inhibiting gas exchange between water, microbial mats, and the atmosphere [11]. Remarkably, most of the genera belonging to the core are part of the rare biosphere as well; likewise, every abundant genus belong to the core. Core functions relative abundances, on the other hand, appear highly conserved between samples (Fig. S15: Supplementary material), with processes such as carbohydrate, amino acid, protein, RNA, and DNA metabolisms being the most abundant ones, unsurprisingly. PCoA ordination method suggest a seasonal pattern of functions, as the dry season samples of 2019 and 2016 were grouped in one cluster, while the rainy season samples of 2016 and 2018 were close to each other in another group (Fig. S15: Supplementary material). The dry season sample of 2017 and the rainy season sample from 2019 does not cluster to any of the aforementioned groups. If interpreted as a stability landscape, the core community do return to the same seasonal stable state despite the press disturbance, unlike the global community. A bigger sample size will determine if these groups do preserve a seasonal pattern or not.

Core co-occurrence networks at the genus level also provide insights into the global core dynamics for both the dry and rainy seasons (Fig. 6b). To begin with, there are six shared clusters of genera in both, dry and rainy seasons. Consistent with the networks built at the phylum level, the rainy season network for the 250 core genera displayed the addition of a new cluster that was not previously present in the dry season network. Additionally, many of the present genera relocate to different clusters between the dry and rainy seasons. This behavior could reflect how the core taxa differentially interact with each other in response to differential environmental pressures. Even though these taxa are present in the whole community, regardless of the season, it is natural to infer that interactions within this core community are the ones that change through the seasons. For both networks, global metrics were calculated, and once again, modularity and positive/negative ratio show consistency with the whole-community phylum networks (Table S13: Supplementary material); dry and rainy seasons displayed relatively high modularity values (0.17 and 0.22, respectively), and the slightly lower value during the dry season could reflect a drop in community stability during this state. Positive edges in both networks account for the $\sim$41%, which result in high positive/negative ratios that further suggest a resistant and resilient community [26, 111]. Finally, hub taxa were predicted for each network, and among them, *Coleofasciculus*, *Chitinispirillum*, *Desulfonatronovibrio*, *Desulfovermiculus*, *Halanaerobium*, *Halomonas*, *Halorhabdus*, and *Halorubrum* are shared hubs between the seasons. Two hub groups appear during the dry season, whereas during the rainy season, every hub genus belong to the same cluster. It seems that some hub taxa (including *Desulfonatronovibrio*, *Coleofasciculus*, *Halanaerobium*, and *Chitinispirillum*) are also involved in the differential interactions between seasons. Given that sample size is small, detailed interaction analysis should be taken with caution. Thus, we restrain ourselves to just an exploratory, non-conclusive, global analysis of these networks.

## Conclusions and Perspectives

Based on our results, we have concluded that the Archean Domes microbial system is able to maintain its stability despite seasonal fluctuations, including prolonged drought periods. This stability can be attributed to the diverse yet resilient-poor community and the functional redundancy that enables the system to resist significant alterations during and after perturbations. To gain a deeper understanding of the microbial dynamics of this system, future studies should focus on tracking its evolution over a longer timespan, measuring more environmental parameters, and increasing the robustness of sampling through replicates. Specifically, future studies should aim to identify the mechanisms through which moderately-abundant and rare biosphere taxa contribute to the system’s stability, confidently identify hub taxa and their interactions, and comprehend the interplay between Archaea and Bacteria. Overall, our findings highlight the importance of understanding the mechanisms that enable microbial systems to maintain stability in response to environmental fluctuations, which can have significant implications for ecosystem functioning and resilience.

Further work on the ecological stability of microbial mats have the potential to aid in our understanding of early life on Earth. Previous studies have suggested that laminae within stromatolites represent successions of microbial communities as a response to environmental fluctuations [119, 120]. This concept applies to non-lithifying microbial mats as well. For instance, colonization-growth-burial dynamics are easily recognized in siliciclastic rocks from the Moodies Group (3.22 Ga) [cf. photographs of polished slabs in 6, 121]; during times of low hydrodynamic energy, microbial mats develop where the deposited sand is recolonized by planktonic or mat-dwelling microbes, after which sandy sediment deposition buries the previous microbial community (i.e., an ecological disturbance). Individual kerogenous laminae derived from the decay of microbial mats most likely represent a snapshot of the microbial community, and could potentially display diversity in geochemical signals such as those reported in organic films from the Buck Reef Chert (3.4 Ga) [122], perhaps inherited from taxonomic turnover and drift of a functionally stable ecosystem. To gain a better understanding of how these systems have responded to environmental perturbations in the past, it is essential to develop mechanistic models that explain microbial dynamics under environmental pressures within modern microbial mats.

### Supplementary Information

Below is the link to the electronic supplementary material.

Supplementary file 1 (pdf 49450 KB)

## Data Availability

Raw reads for all samples can be found at the NIH BioProject: Cuatro Cienegas Basin, Archaean Domes, under the accession number PRJNA847603 (https://www.ncbi.nlm.nih.gov/sra/PRJNA847603). This study comprises SRA accessions SRX15887985-SRX15887990.
