# Supplementary material for: A Metagenomic Time-Series Approach to Assess the Ecological Stability of Microbial Mats in a Seasonally Fluctuating Environment
Source: Microb Ecol. 2023 Jul 2;86(4):2252–70. doi: 10.1007/s00248-023-02231-9 (PMC10640475; doi:10.1007/s00248-023-02231-9)
Supplement: Supplementary file 1 — (pdf 49450 KB) [file 248_2023_2231_MOESM1_ESM.pdf]

A Metagenomic Time-Series Approach to Assess the Ecological Stability of Microbial Mats in a Seasonally Fluctuating Environment

Supplementary material

Supplementary figures

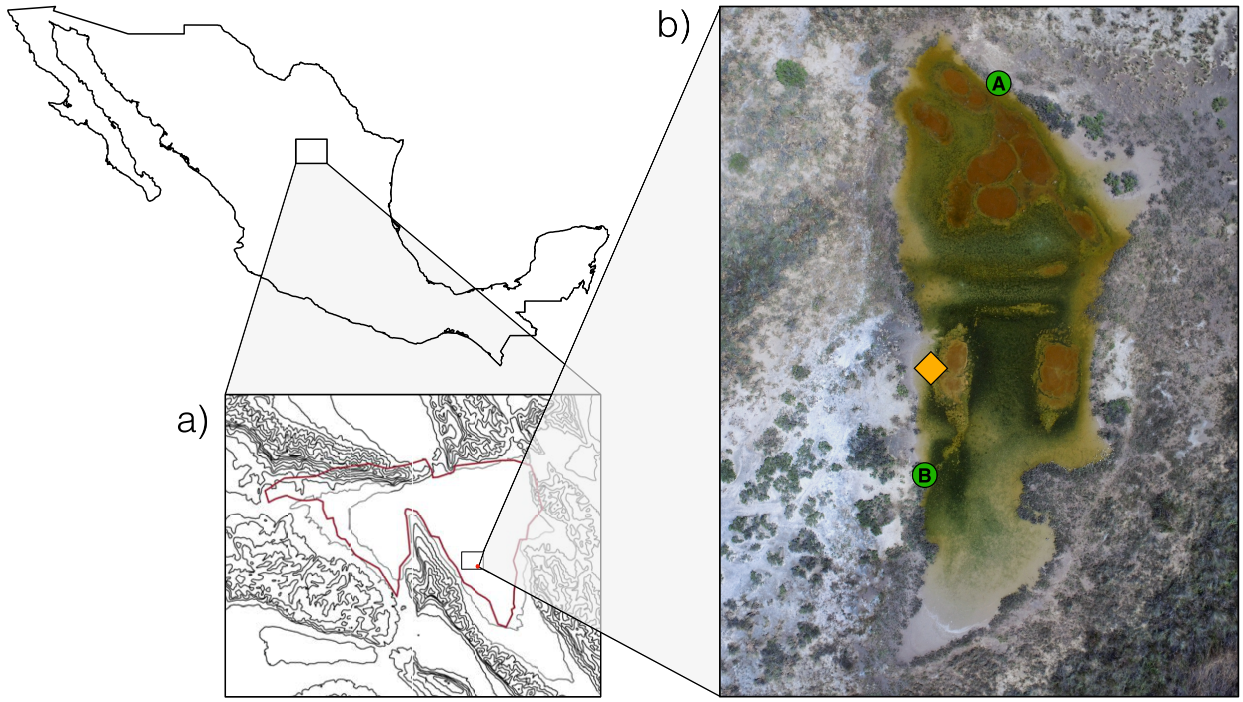

Fig. S1: Overview of the Archean Domes sample site. *a)* the Cuatro Ciénegas Basin, located in the Chihuahuan Desert. Depicted as a small rectangle within the basin, the Pozas Azules ranch where the Archean Domes is located. *b)* Aerial view of the Archean Domes pond during the september 2019 sampling. The pond is roughly 50x25 m. The yellow diamond show the sampling point for the six metagenomes studied in this work. Green circles, A and B, indicate the picture location from Fig. 1a and 1b, respectively. Panel *a)* was built using the public sources CONANP 664 (http://sig.conanp.gob.mx/website/pagsig/mapas_serie.htm) and CONABIO SNIB 665 (http://www.conabio.gob.mx/informacion/gis/). Photo credit for *b)*: David Jaramillo.

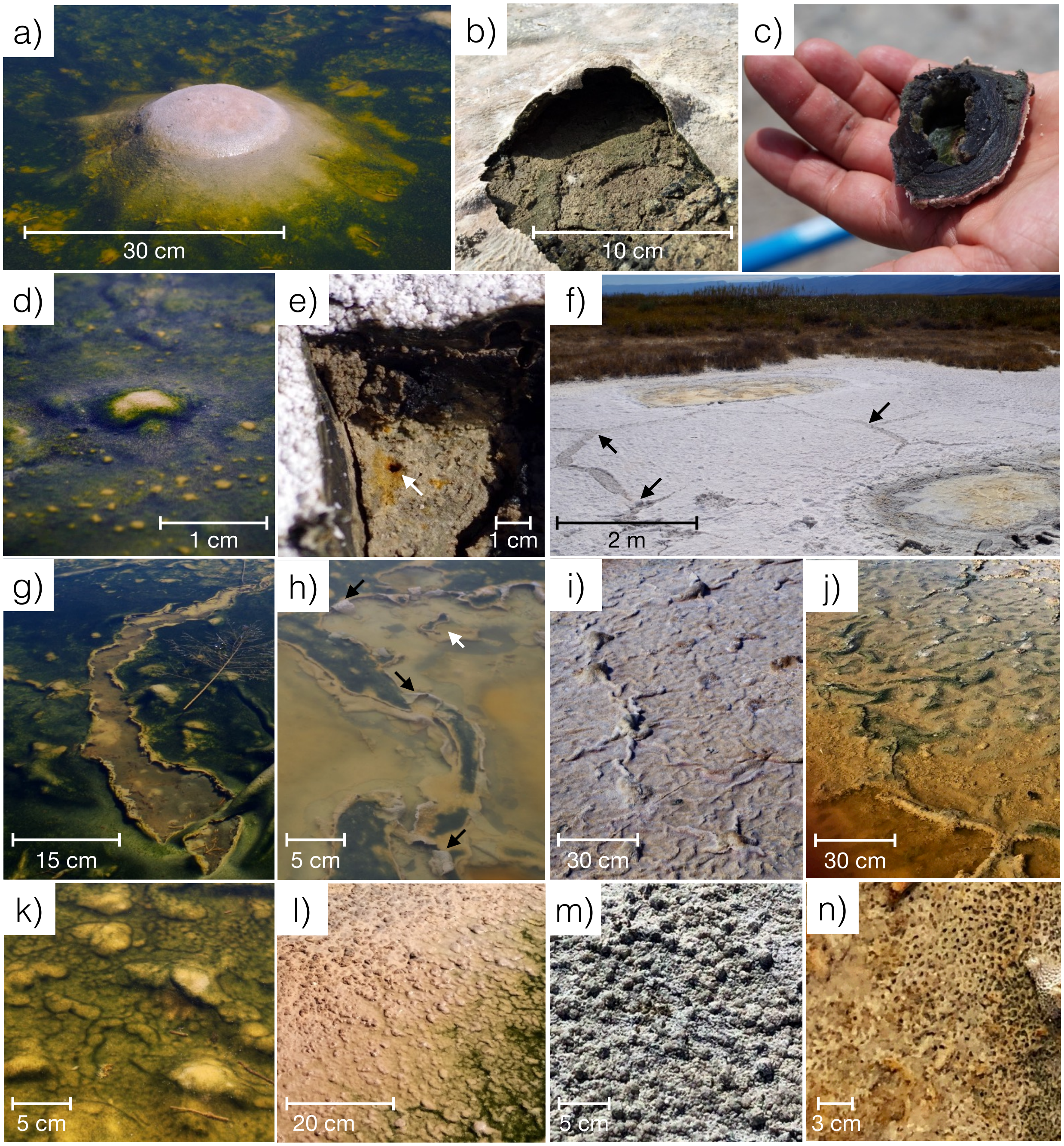

Fig. S2: Microbially Induced Sedimentary Structures (MISS) found at the Archean Domes system. Gas dome structures are the most distinctive feature during both *a)* rainy, and *b)* dry seasons. *c)* During the dry season, most gas domes are uncollapsed and retain their shape. *d)* Small sized gas dome alongside milimiter sized blisters. e) Potential gas duct exposed during sampling (arrow). *f)* Polygonal, meter-sized shrinkage cracks; angular intersections between cracks can be observed (arrows). *g-h)* Shrinkage cracks with instances of rolled-up structures (black arrows) and mat chips (white arrow). *i-j)* Elongated petee structures. *k)* Small-scale reticulated mat surface. *l)* Cauliflower shaped nodules at the edge of the pond. *m)* Trapped and, *n)* disrupted milimeter-sized gas bubbles.

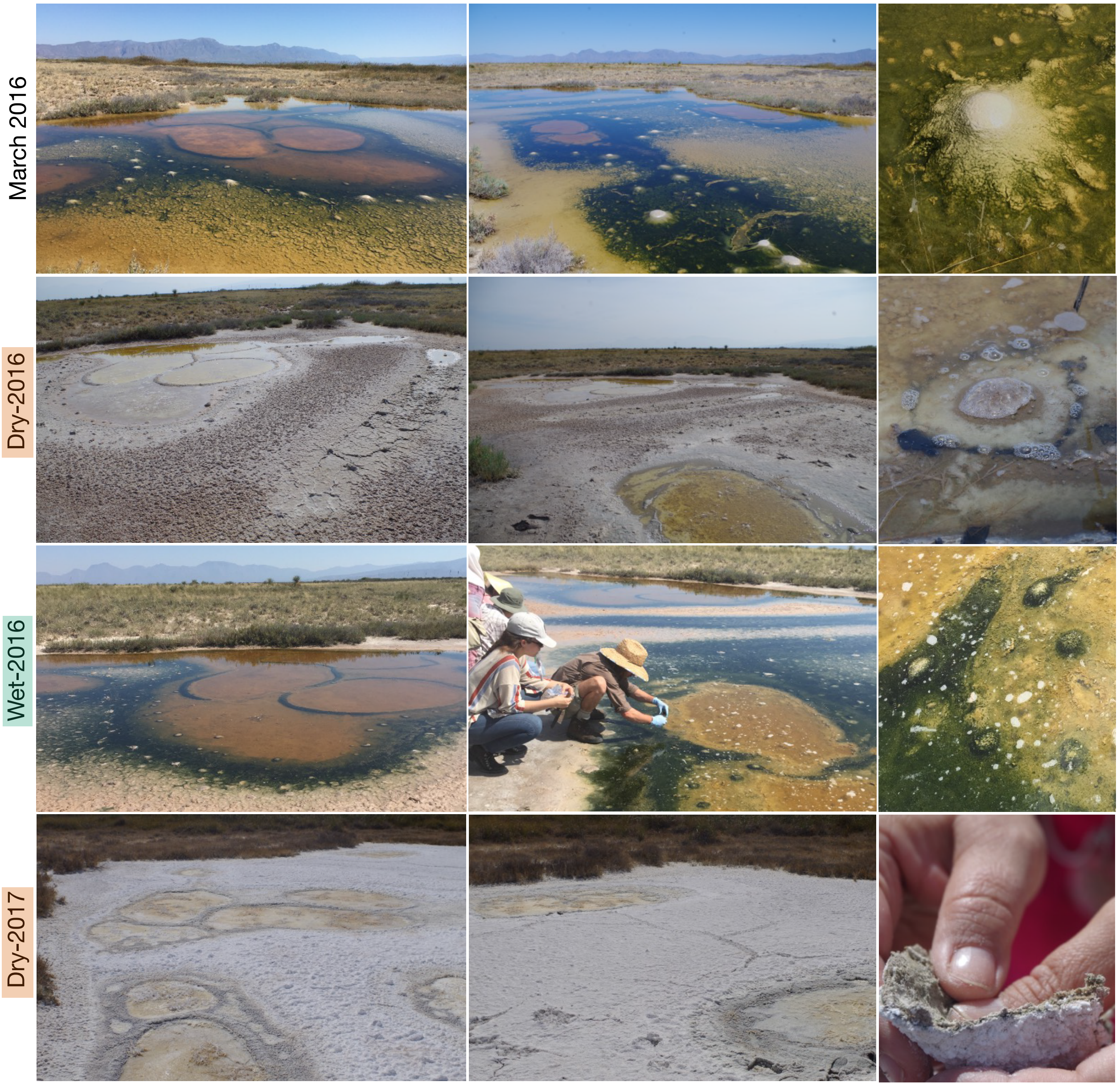

Fig. S3: Photo gallery of the Archean Domes during each of the samplings included in this study. On the right side, gas domes features from the respective sampling for comparison. ID Labels are described in the main text. March 2016 and September 2021 photos are included for comparison only, since no samples from those times were included for this study. Photos from September 2021 show the increase in water level, salt dissolution, and green mat development after a slight rainfall.

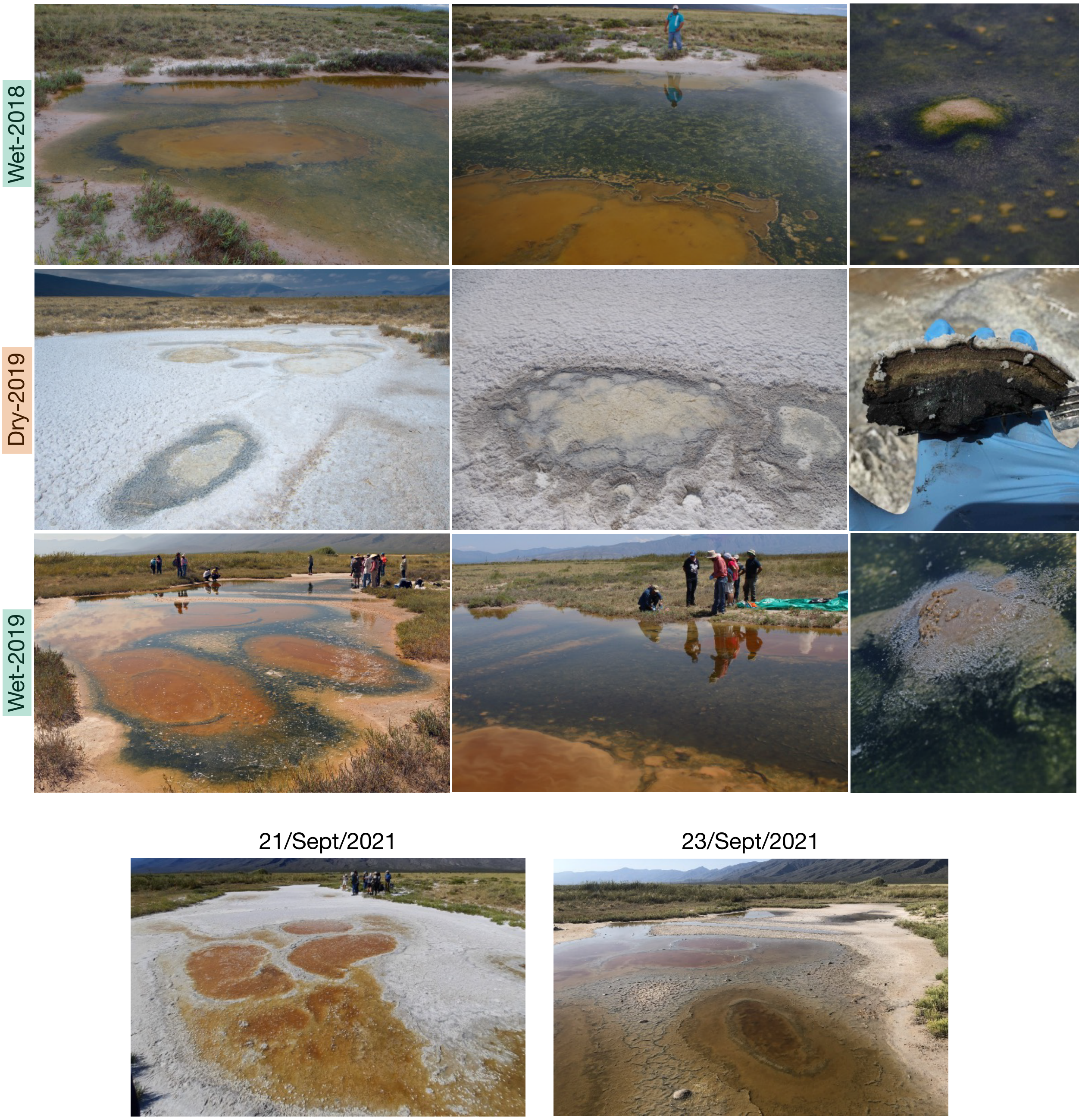

Fig. S3 (continued)

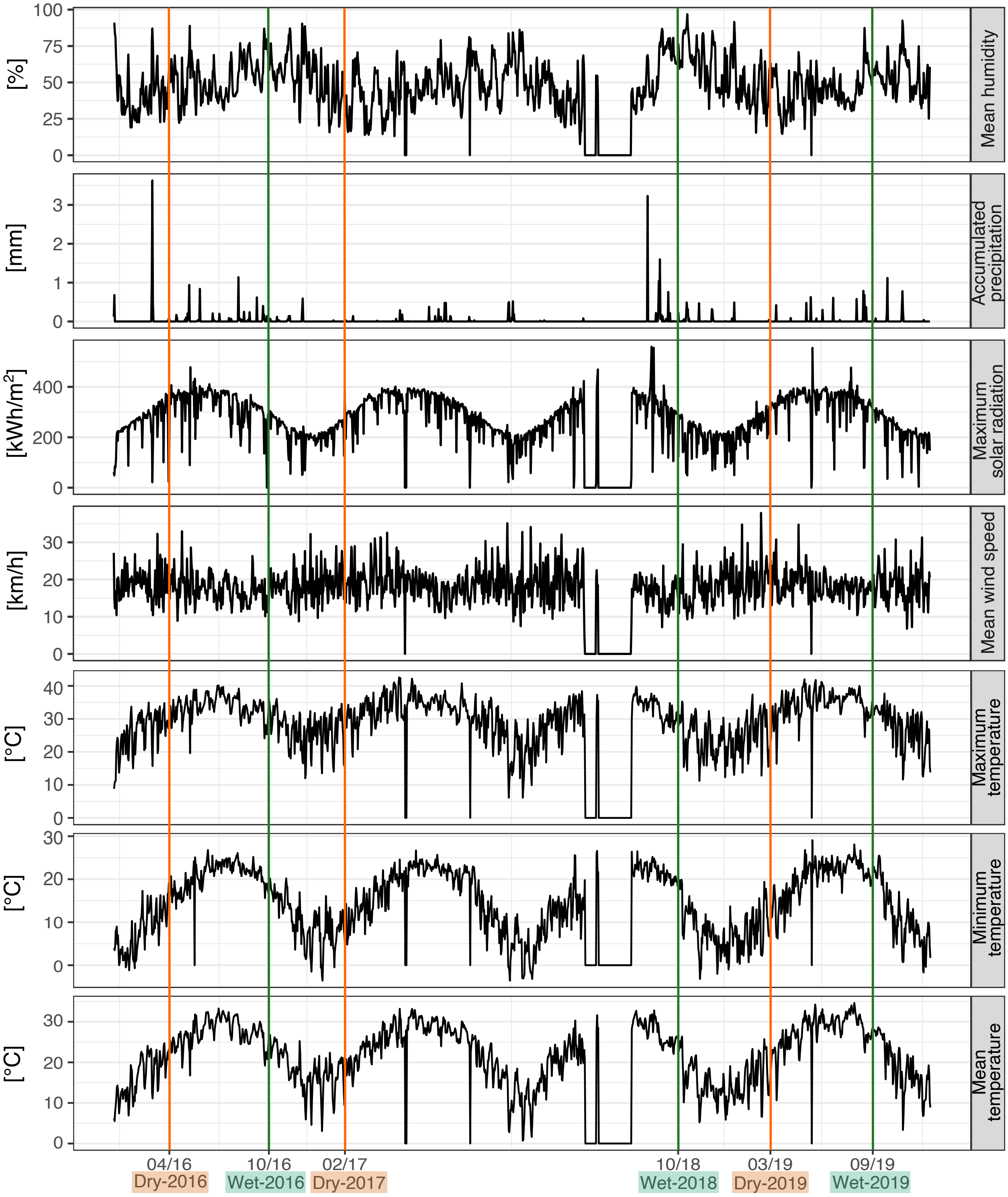

Fig. S4: Weather data retrieved from the EMA weather station No. 15DBB372, Cuatro Ciénegas, from 2016 to 2019 (https://smn.conagua.gob.mx/es/). Data gaps during some months of 2018 represent that service was unavailable at that time. Lines show the sample day variables for each sample. Colors indicate rainy (green) and dry (orange) seasons.

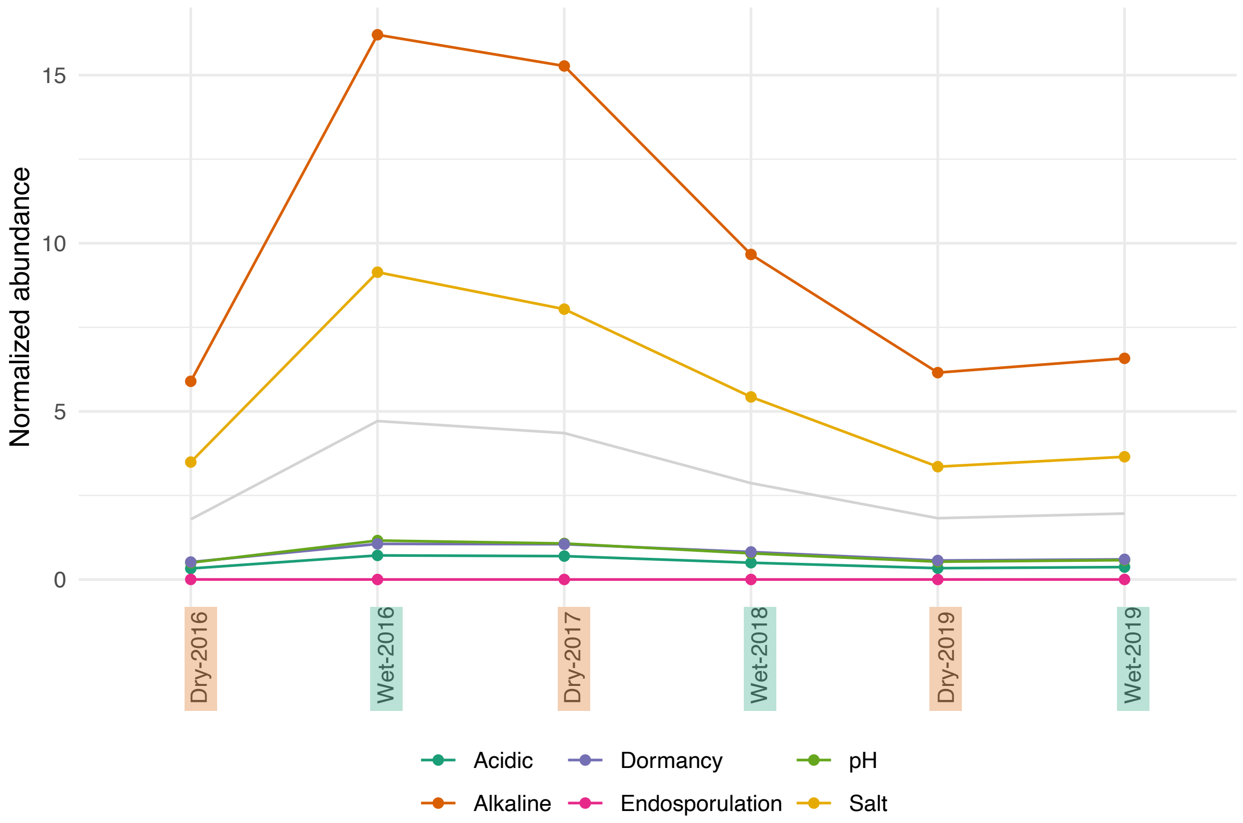

Fig. S5: Resistance genes found at the Archean Domes. Through all samples, most resistance genes are associated to alkaline and salt response genes. The gray line shows the mean normalized abundance of stress response genes for each sample. Selected GO terms for each category are shown in Table S9.

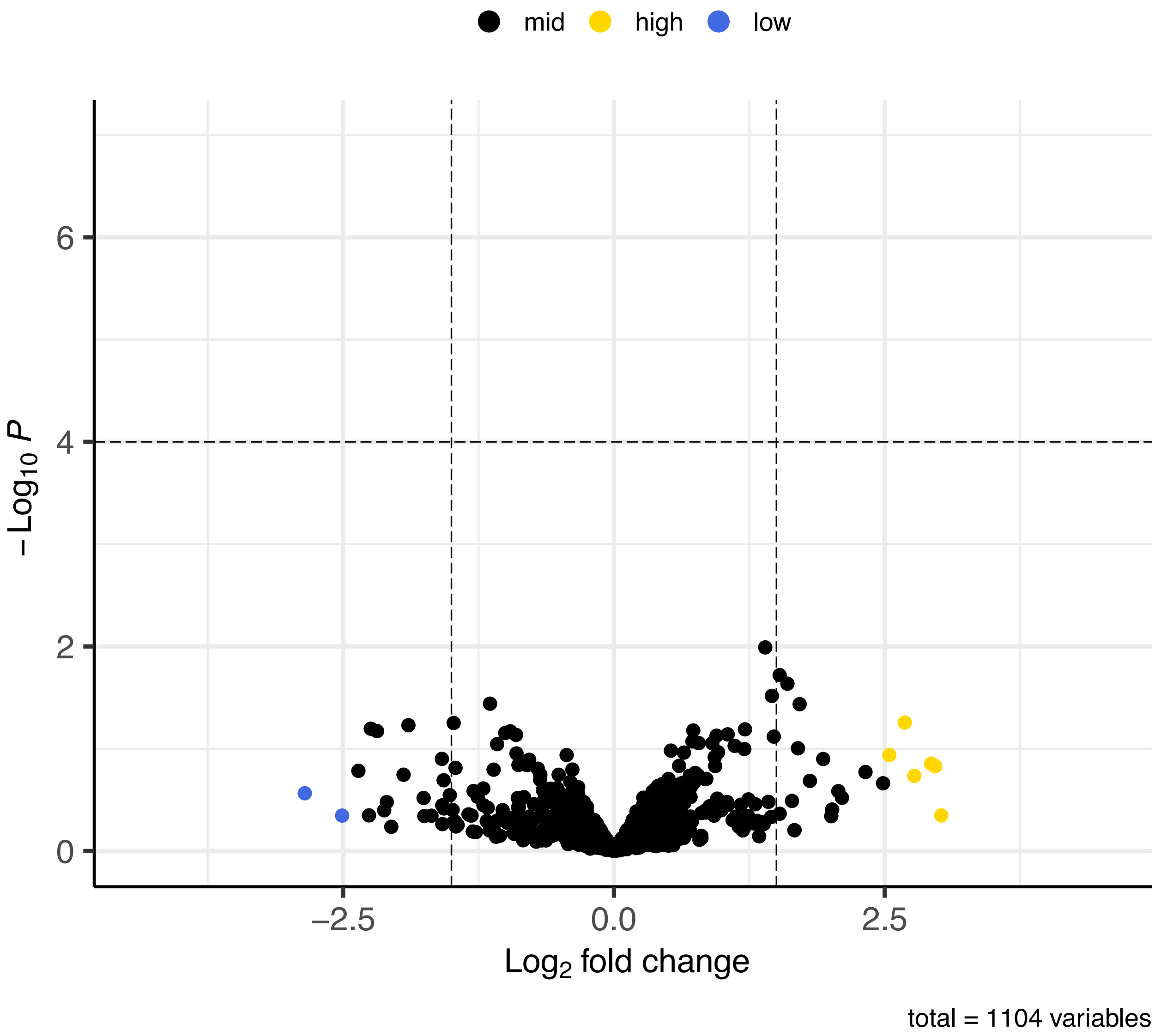

Fig. S6: Differential expression analysis of fuctions adapted to a metagenomic study (gene functions). No statistical significance was found for each function, although there are slight increases for some functions in both rainy and dry seasons (discussed in the main text). black points (mid) show Log_2 Fold Change $< |2.5|$, which are interpreted as constant between seasons. 1104 functions were used for this analysis.

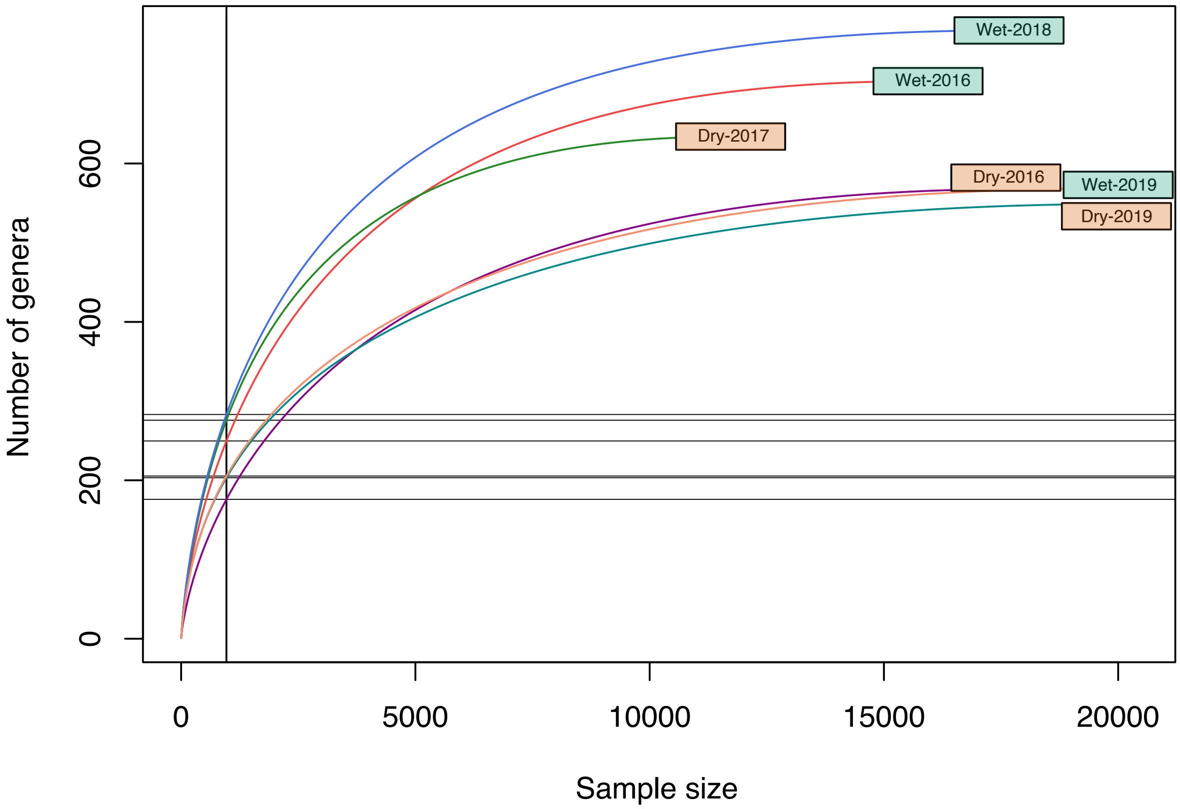

Fig. S7: Rarefaction curves for each sample studied. Sample size and number of genera are depicted in the horizontal and vertical axes, respectively. Each sample reaches saturation of genera richness and is suitable for sample comparison.

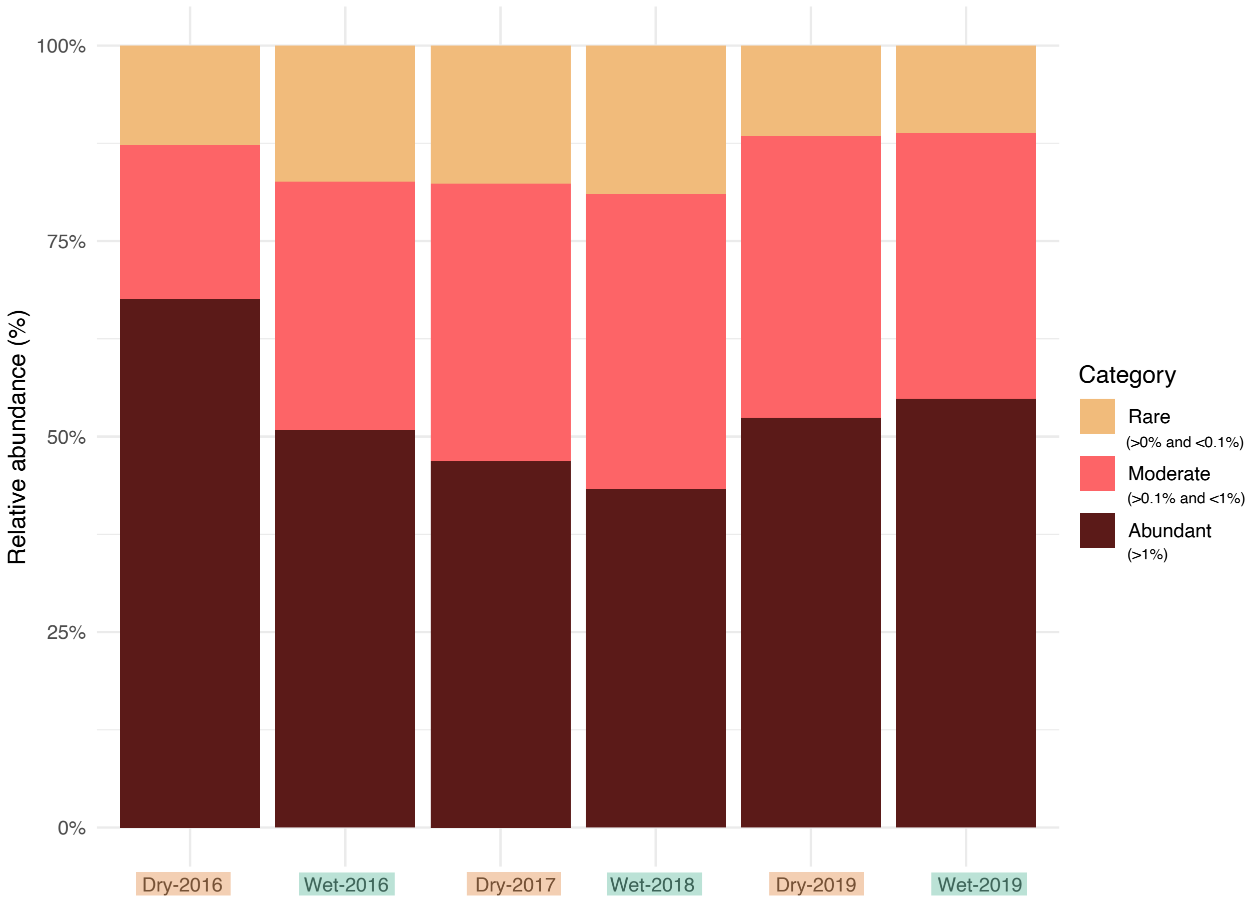

Fig. S8: The Archean Domes community based on their relative abundance at genus level. Abundant taxa comprise most of the whole community, in contrast to moderate and rare taxa abundance.

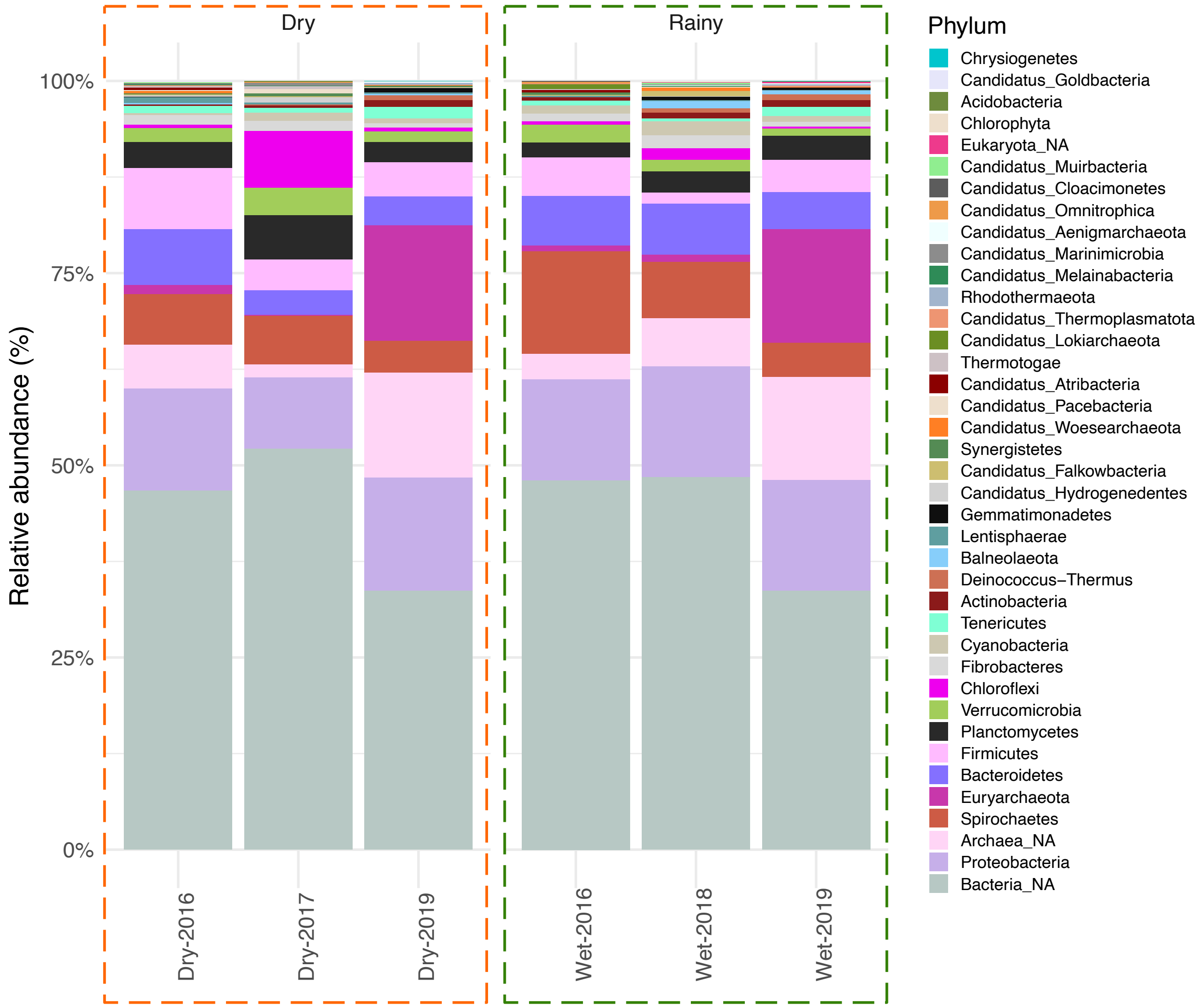

Fig. S9: Ribosomal-protein taxonomic profile of the Archean Domes system. Not annotated sequences were grouped in the NA categories. Selected ribosomal-protein families for this taxonomic annotation are described in the main text.

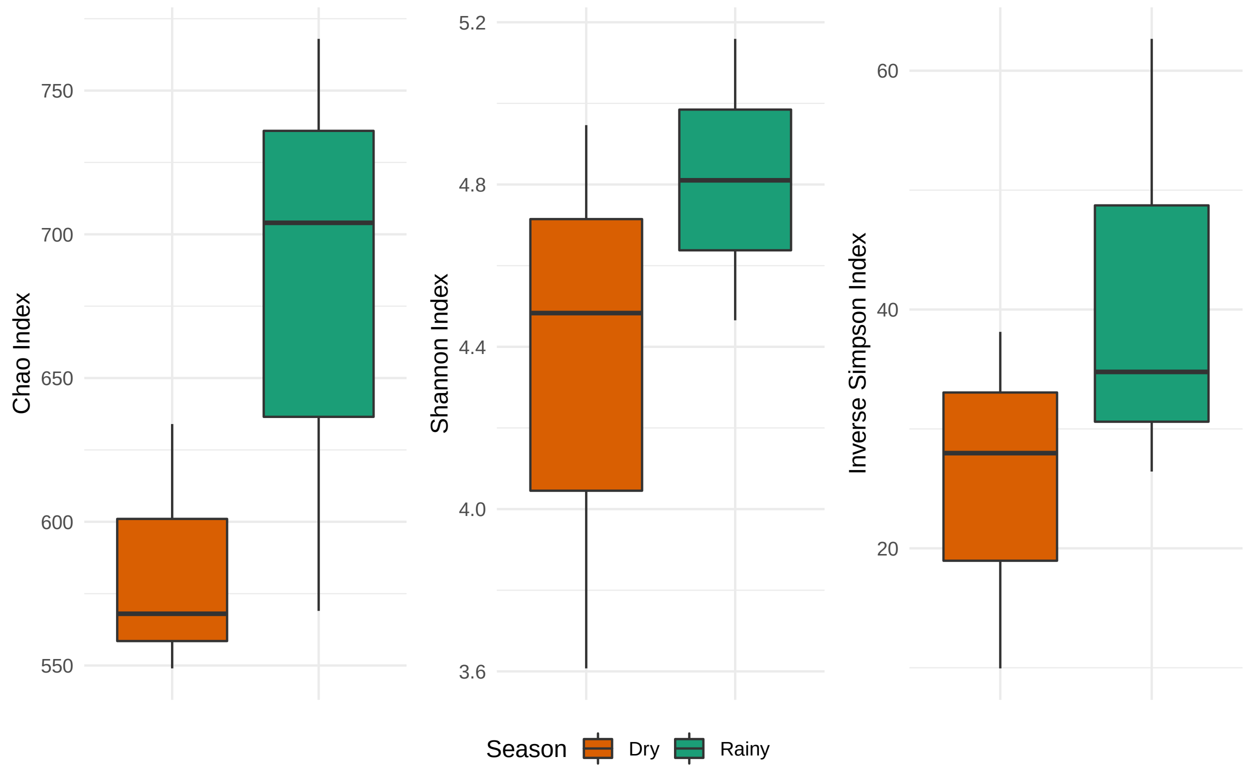

Fig. S10: Boxplots for diversity comparison for each seasonal state. Performing Wilcoxon Rank Sum test showed no statistical differences between seasonal diversity.

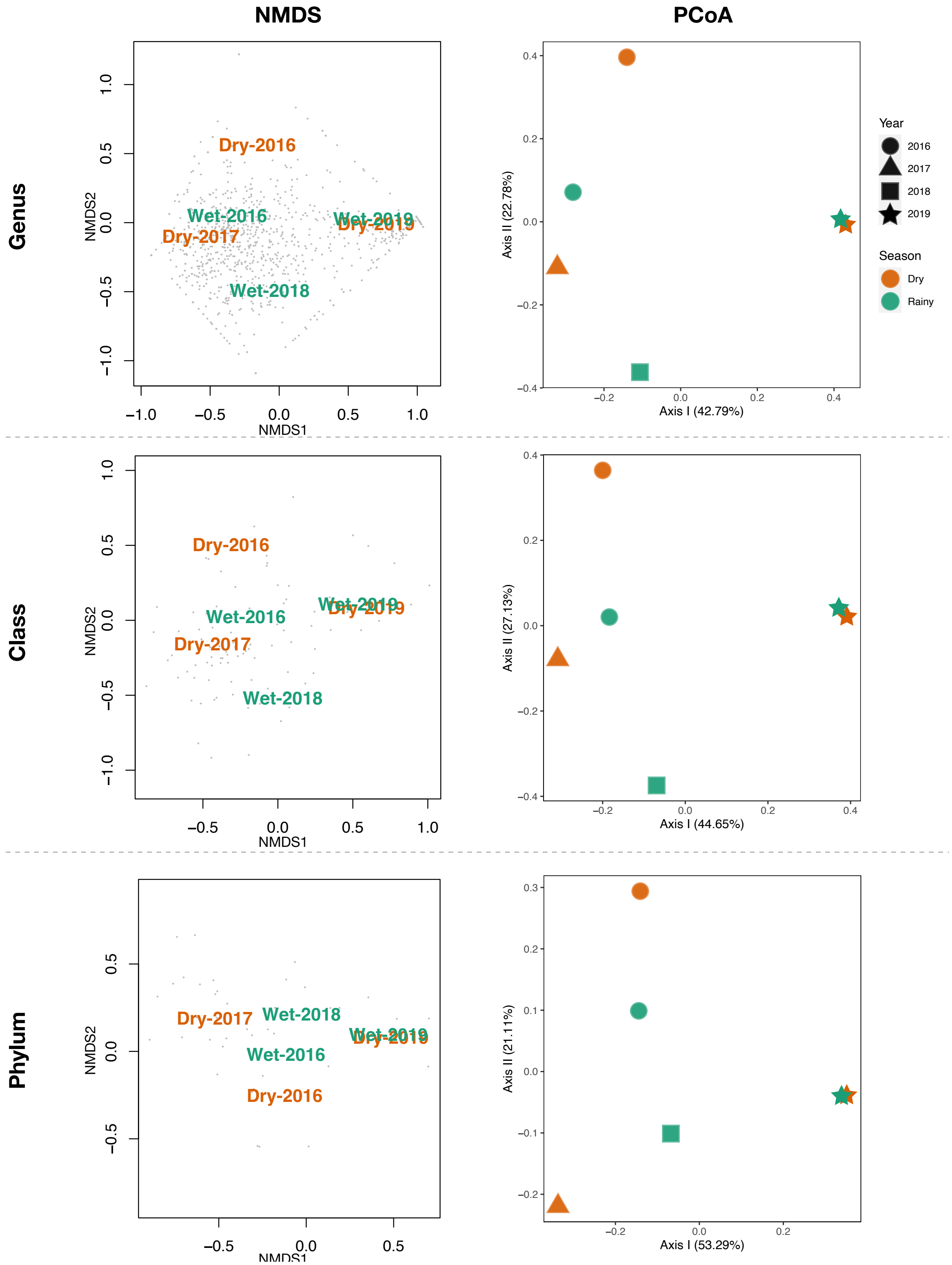

Fig. S11: NMDS (Left) and PCoA (Right) analyses at the genus (top), class (middle), and phylum (bottom) level with Bray-Curtis measure. Both analyses showed no seasonal aggregation.

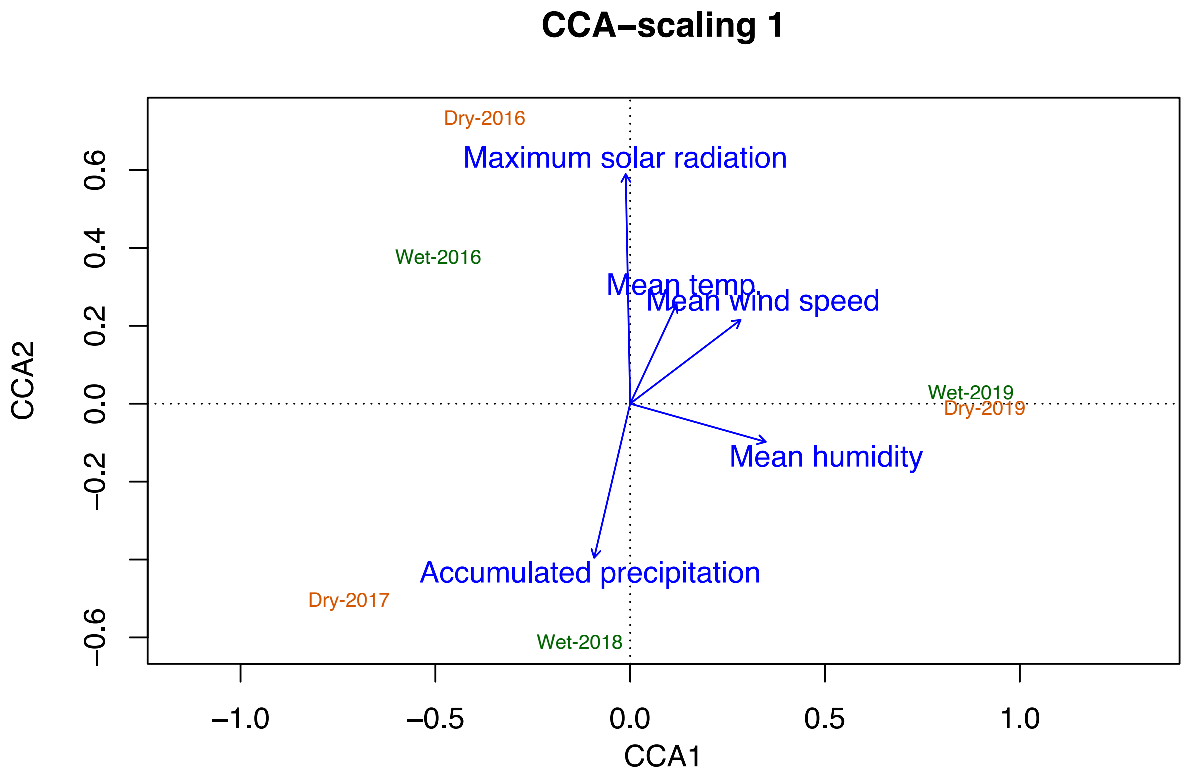

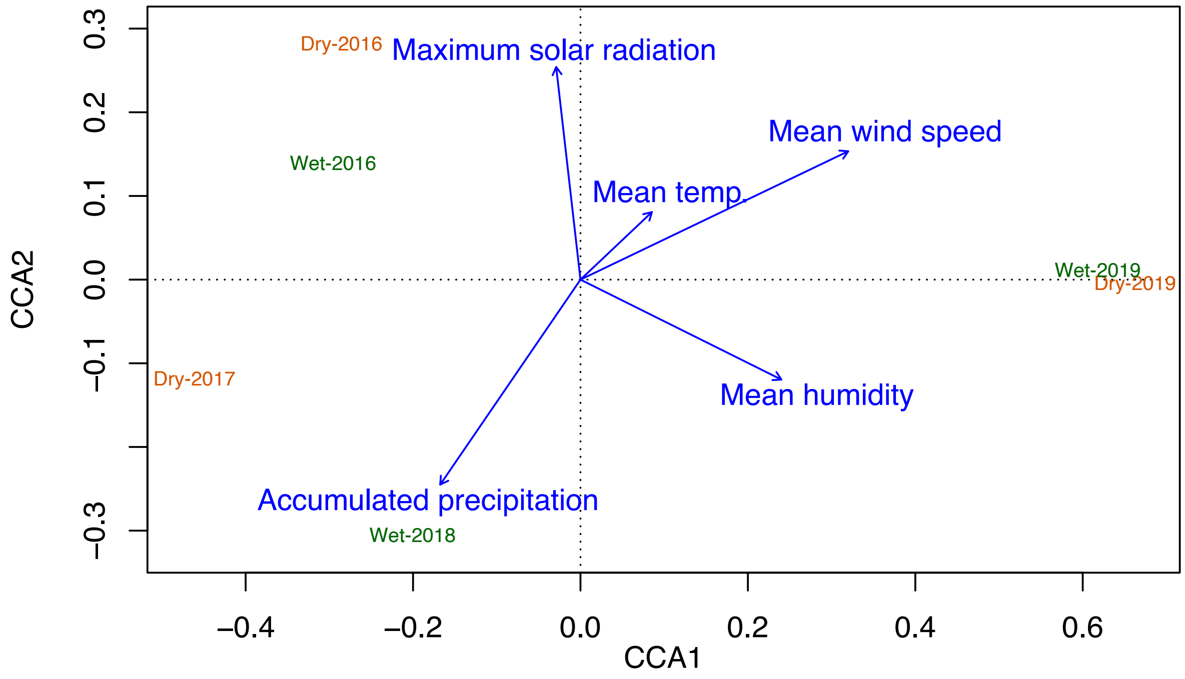

Fig. S12: CCA analysis at the genus (top) and phylum (bottom) level with the environmental data provided by the EMA meteorlogical station. Meteorlogical data mean values for each month of sampling were used as input.

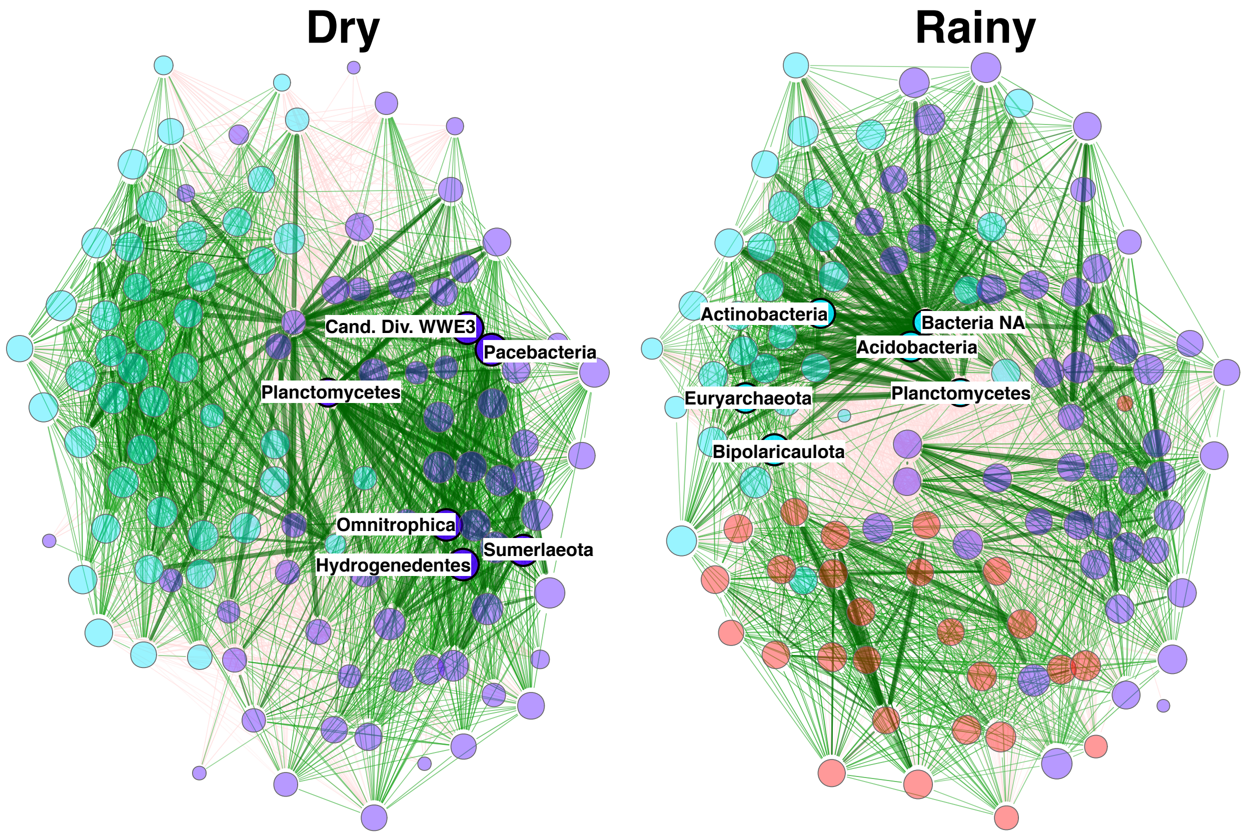

Fig. S13: Global network of shared phyla between seasons. Networks were built with the top 120 phyla across samples. Colors show clusters, and the same colors in both networks correspond to the same structural cluster. It can be appreciated the addition of a new cluster (red) during the rainy season. Green edges represent positive relationships, while red edges represent negative ones.

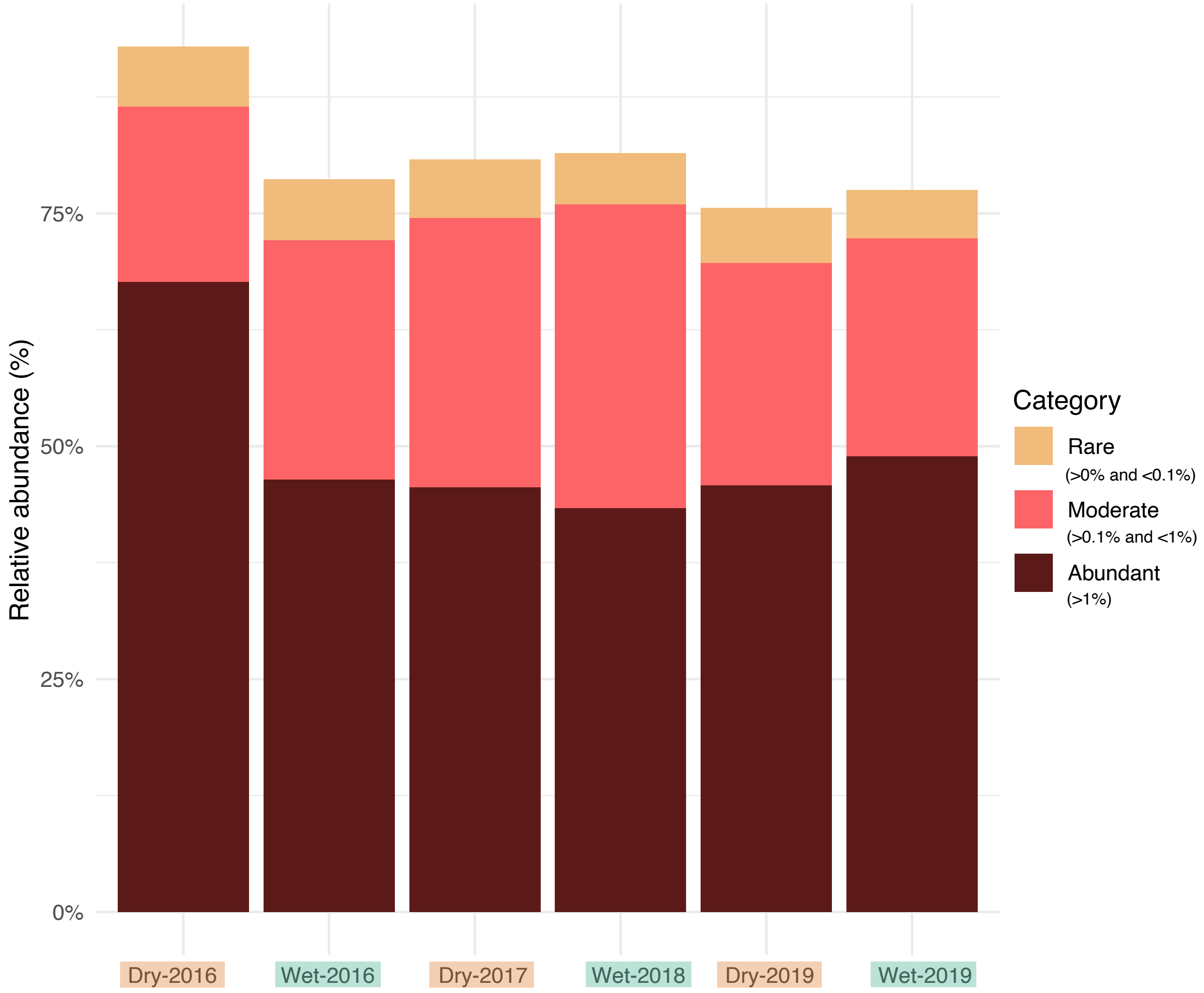

Fig. S14: Relative abundance of the Archean Domes core community at the genus level. Abundant taxa comprise most of the core, in contrast to moderate and rare taxa abundance.

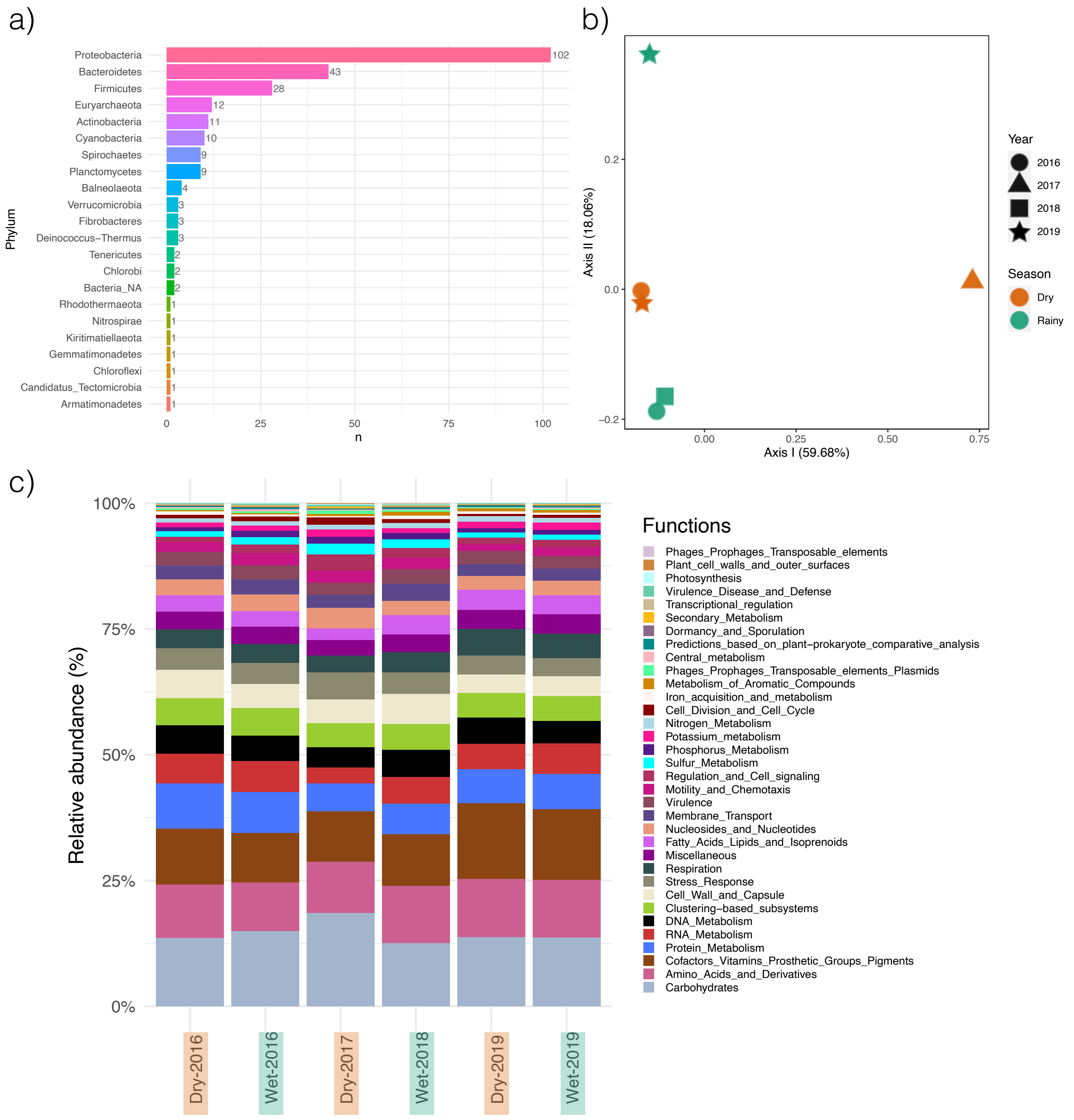

Fig. S15: Core taxonomic and functional details. *a)* Core composition at phylum level. The number of genera for each phylum appears next to each bar. *b)* Core functions under a PCoA ordination method. Roughly, there appears to be a seasonal pattern with a close association between two dry and rainy season samples, although the rainy sample from 2019 and the dry sample from 2017 does not group with any other sample. *c)* Function composition for core genera. Overall, similar relative abundances between samples can be appreciated.

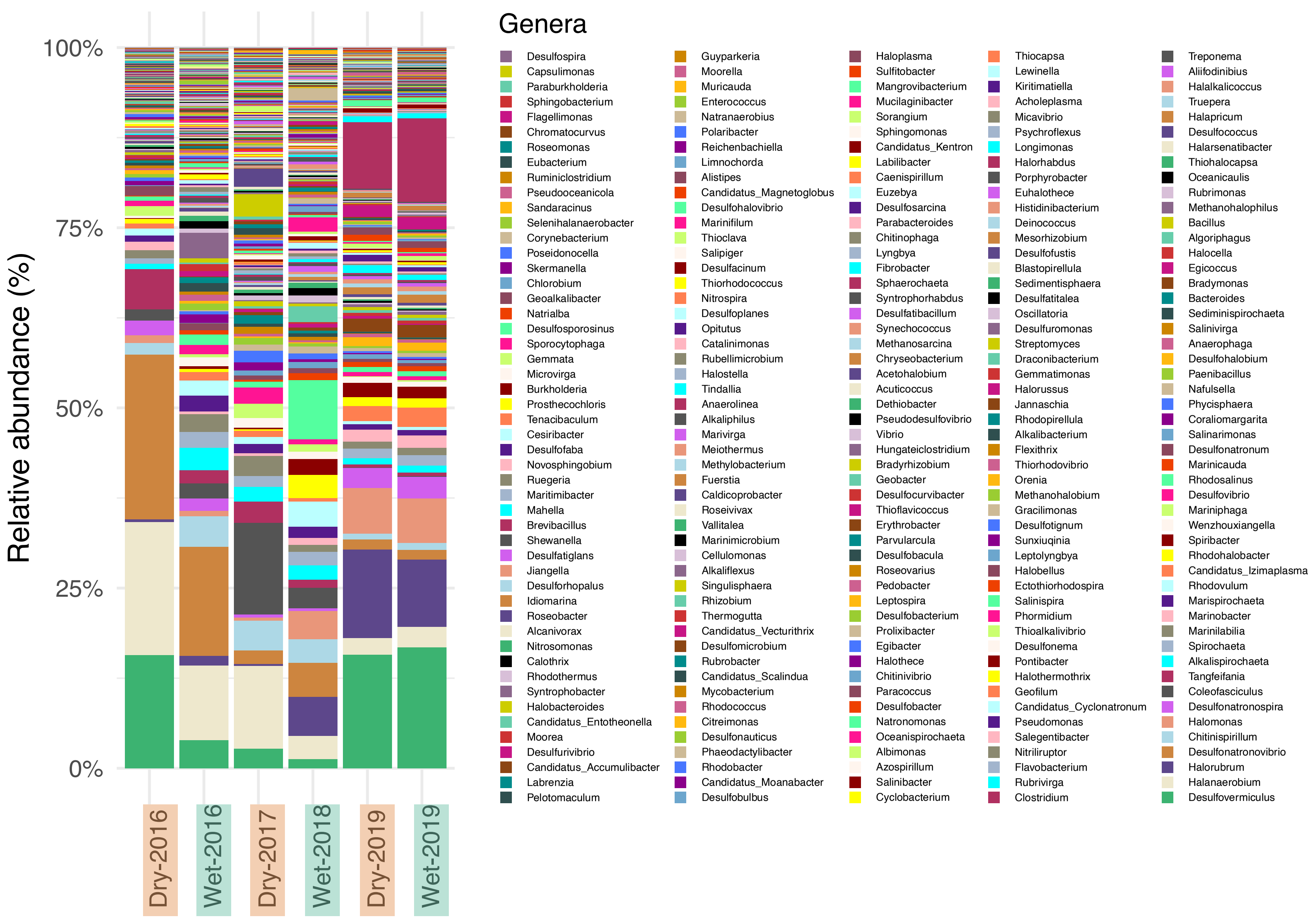

Fig. S16: Core composition at the genus level. Each of the 250 genera that constitute the core is shown in order to emphasize the changes in relative abundance for each sample.

Supplementary tables

Table S1: Methane (CH_4) and carbon dioxide (CO_2) concentrations inside the gas domes as measured in the samplings of April 2016 and February 2017. No CO_2 was measured for April 2016.

Sampling point	CH_4 concentration (μg/L)		CO_2 concentration (mg/L)	
	April 2016	February 2017	April 2016	February 2017
1	2.6 ± 1.0	193.2 ± 133.7	NA	1.40 ± 0.08
2	15.2 ± 1.2	102.1 ± 18.4	NA	1.08 ± 0.05
3	19.6 ± 1.4	402.2 ± 209.5	NA	1.13 ± 0.17

Table S2: Carbon, nitrogen, and phosphorus content for dry and rainy 2019 samples (March 2019 and September 2019, respectively). Sediment pH and Grain size analysis are also shown.

Sampling point (March 2019)	pH	Organic matter (%)	Organic carbon (mg/g)	Total nitrogen (mg/g)	Organic nitrogen (mg/g)	Inorganic nitrogen (mg/g)	Total phosphorus (mg/g)	Inorganic phosphorus (mg/g)	Organic phosphorus (mg/g)	Sand (%)	Silt (%)	Clay (%)	Texture class
1	8.25	8.3	63.96	4.03	2.85	1.18	10.3	9.41	0.89				
2	8.46	6.27	48.36	3.52	1.84	1.68	7.53	6.3	1.23				
3	8.44	7.69	59.28	4.87	2.92	1.95	13.75	11.41	2.34				
4	8.4	7.69	59.28	5.47	4.26	1.21	7.53	6.52	1.01				
5	8.65	4.25	32.76	6.1	2.25	3.85	9.64	9.19	0.45	68.3	23.44	8.5	Sandy loam
6	8.41	6.88	53.04	4.45	2.01	2.44	10.3	9.08	1.22				
7	8.44	7.49	57.72	2.68	2.18	0.5	7.41	6.19	1.22				
8	8.64	8.11	62.4	6.7	5.2	1.5	9.3	8.74	0.56				
Average	8.46125	7.085	54.6	4.7275	2.93875	1.78875	9.47	8.355	1.115				
SD	0.130650843	1.318353952	10.1442848	1.339506839	1.194456691	1.011398431	2.12215524	1.855285885	0.579137289				

Sampling point (September 2019)	pH	Organic matter (%)	Organic carbon (mg/g)	Total nitrogen (mg/g)	Organic nitrogen (mg/g)	Inorganic nitrogen (mg/g)	Total phosphorus (mg/g)	Inorganic phosphorus (mg/g)	Organic phosphorus (mg/g)	Sand (%)	Silt (%)	Clay (%)	Texture class
1	8.45	5.1	39	2.7	2.5	0.2	29.69	27.52	2.17				
2	8.28	4.66	35.88	2.7	2.5	0.2	51.29	39.63	11.66				
3	8.74	4.05	31.2	2.7	2.5	0.2	46.97	36.6	10.37	66.1	24	9.9	Sandy loam
4	8.32	3.64	28.08	2.7	2.5	0.2	50.43	37.47	12.96				
Average	8.4475	4.3625	33.54	2.7	2.5	0.2	44.595	35.305	9.29				
SD	0.208066496	0.646032765	4.85023711	—	—	—	10.1105539	5.344034057	4.863010042				

Table S3: Sample-day weather parameters from EMA weather station No. 15DBB372, Cuatro Ciénegas, Coahuila, Mexico.

Sample/parameter	Dry-2016	Wet-2016	Dry-2017	Wet-2018	Dry-2019	Wet-2019
Sample day/time	2016/04/09 9:48:38	2016/10/04 12:02:35	2017/02/17 11:38:28	2018/10/06 9:10:20	2019/03/20 11:32:19	2019/09/19 12:53:19
Mean wind direction (°)	153.5	149.5	245	170.5	123.5	111.5
Mean wind speed (km/h)	5.55	5.1	15.45	14.15	23.9	36.25
Max. wind speed (km/h)	14.3	12.1	43	22.5	39.1	63.2
Min. Temperature (°C)	23.1	28.1	21.1	24.6	16.8	29.5
Mean Temperature (°C)	23.9	29.2	22.53	25.13	17.3	29.73
Max. Temperature (°C)	24.8	29.8	23.1	25.9	17.9	29.9
Mean humidity (%)	63.83	50.5	28.5	70	45.67	54.15
Mean pressure (mbar)	923.52	925.07	927.82	928.43	935.37	929.6
Max. solar radiation (kWh/m^2)	721	859	864	607	971	942

Table S4: Raw reads and quality control (QC) metadata for each shotgun metagenome studied.

Sample/ parameter	Raw reads	Low QC reads	Total reads	Paired reads	Forward Unpaired	Reverse Unpaired
Dry-2016	28,859,454	2,425,863	26,433,591	21,012,271	4,755,240	666,080
Wet-2016	4,772,053	287,632	4,484,421	3,722,418	671,964	90,039
Dry-2017	8,203,484	790,818	7,412,666	5,915,153	1,255,747	241,766
Wet-2018	10,030,782	1,561,639	8,469,143	6,115,153	2,098,789	255,201
Dry-2019	25,873,990	3,589,352	22,284,638	17,676,424	3,645,559	962,655
Wet-2019	20,153,088	1,471,902	18,681,186	16,378,340	1,784,198	518,648

Table S5: Metagenome assembly metadata for each sample. Minimum, average, and maximum contig length is also shown

Sample/ parameter	Total base pairs	Assembled (bp)	Not assembled (bp)	Min. contig length (bp)	Avg. contig length (bp)	Max. contig length (bp)
Dry-2016	9,838,571,624	857,058,612	8,981,513,012	500	1,274.80	159,881
Wet-2016	1,491,700,706	190,580,164	1,301,120,542	500	1,243.70	86,189
Dry-2017	2,627,434,540	304,829,428	2,322,605,112	500	1,291.70	121,677
Wet-2018	2,985,543,837	329,842,768	2,655,701,069	500	1,089.90	75,330
Dry-2019	7,799,883,462	588,197,199	7,211,686,263	500	1,142.20	142,626
Wet-2019	7,744,171,739	621,571,725	7,122,600,014	500	1,108.90	124,235

Table S6: Metagenome assembly metadata for sequences not initially assembled.

Sample/ parameter	Assembled sequences	Not assembled sequences (Forward)	Not assembled sequences (Reverse)
Dry-2016	672,295	1,263,688	197,513
Wet-2016	153,232	244,559	26,150
Dry-2017	235,985	323,183	66,130
Wet-2018	302,633	735,840	91,703
Dry-2019	515,141	888,820	242,019
Wet-2019	560,549	427,091	121,222

Table S7: Taxonomic annotation (CAT) metadata for each sample and relative abundances for each superkingdom.

	Reads processed				Relative abundance (%)			
Sample/parameter	Total Found	Unclassified	Classified	Phylotypes	Archaea	Bacteria	Eukaryota	Viruses
Dry-2016	1,562,120	1,083,248	478,872	8,699	3.06	96.65	0.20	0.08
Wet-2016	1,288,875	578,442	710,433	10,969	4.27	95.45	0.17	0.11
Dry-2017	285,524	190,940	94,584	3,872	1.44	98.38	0.13	0.05
Wet-2018	1,791,166	867,900	923,266	12,370	7.27	92.01	0.51	0.21
Dry-2019	1,833,943	1,020,352	813,591	10,079	33.61	65.83	0.19	0.36
Wet-2019	2,142,378	884,359	1,258,019	11,840	33.60	65.83	0.19	0.39

Table S8: Relative abundances for each superkingdom based on ribosomal-protein annotation. Selected ribosomal-protein families for this taxonomic annotation are described in the main text.

	Relative abundance (%)		
Sample/ superkingdom	Archaea	Bacteria	Eukaryota
Dry-2016	7.36	92.55	0.09
Wet-2016	4.83	95.17	0.00
Dry-2017	1.77	98.23	0.00
Wet-2018	7.80	92.02	0.18
Dry-2019	28.98	70.97	0.05
Wet-2019	28.45	71.40	0.15

Table S9: Selected GO terms for specific resistance mechanisms. UniProt amino acid sequences tagged with one or more of the specified GO terms were downloaded for further analysis.

Condition	GO terms
Acidic pH	Response to acidic pH<br>Cellular response to acidic pH<br>Cellular stress response to acidic pH
Alkaline pH	Response to alkaline pH<br>Cellular response to alkaline pH<br>Regulation of cellular response to alkaline pH
pH (general)	Response to pH<br>Cellular response to pH<br>Filamentous growth of a population of unicellular organisms in response to pH
Endosporulation	Endosporulation<br>Myxospore formation<br>Exosporium<br>Microsporidian-type exospore
Dormancy	Dormancy<br>Dormancy process<br>Entry into dormancy
Salt	Response to salt stress<br>Cellular response to salt stress<br>Regulation of response to salt stress<br>Positive regulation of response to salt stress

Table S10: Relative abundances (%) of functions predicted with SUPER-FOCUS.

Function	Dry-2016	Wet-2016	Dry-2017	Wet-2018	Dry-2019	Wet-2019
Carbohydrates	14.423	14.761	16.005	14.599	13.605	13.746
Amino_Acids_and_Derivatives	11.606	11.462	11.498	11.709	12.512	12.413
Protein_Metabolism	9.36	9.32	8.97	8.562	8.544	8.706
Cofactors,Vitamins,Prosthetic_Groups,Pigments	9.263	8.81	8.219	9.717	12.586	12.431
DNA_Metabolism	6.338	6.128	6.023	6.238	5.335	5.22
Clustering-based_subsystems	5.199	5.601	5.511	5.638	4.615	4.597
Cell_Wall_and_Capsule	5.101	4.548	4.676	4.502	3.318	3.281
RNA_Metabolism	5.089	4.898	4.692	4.595	5.538	5.778
Stress_Response	4.177	3.957	3.975	3.764	3.401	3.473
Respiration	3.682	3.683	3.805	3.673	4.218	4.169
Miscellaneous	3.494	3.554	3.394	3.517	4.202	4.127
Nucleosides_and_Nucleotides	3.191	3.265	3.119	2.994	3.265	3.312
Virulence	2.934	2.943	3.101	2.994	2.578	2.621
Fatty_Acids,Lipids,and_Isoprenoids	2.662	2.77	2.793	3.22	3.893	3.817
Membrane_Transport	2.263	2.522	2.554	2.55	2.084	2.027
Motility_and_Chemotaxis	2.083	2.192	1.889	1.652	1.336	1.422
Sulfur_Metabolism	1.508	1.572	1.791	1.623	1.221	1.211
Regulation_and_Cell_signaling	1.193	1.325	1.274	1.529	1.174	1.146
Nitrogen_Metabolism	1.071	1.035	1.087	1.015	1.229	1.166
Phosphorus_Metabolism	1.041	1.159	1.214	1.134	1.164	1.149
Cell_Division_and_Cell_Cycle	0.936	1.041	1.003	0.921	0.797	0.784
Potassium_metabolism	0.623	0.629	0.588	0.657	0.87	0.871
Iron_acquisition_and_metabolism	0.407	0.359	0.35	0.364	0.304	0.311
Phages,Prophages,Transposable_elements,Plasmids	0.379	0.372	0.338	0.381	0.319	0.332
Metabolism_of_Aromatic_Compounds	0.351	0.375	0.44	0.616	0.479	0.444
Central_metabolism	0.339	0.364	0.378	0.364	0.354	0.396
Predictions_based_on_plant-prokaryote_comparative_analysis	0.334	0.37	0.355	0.436	0.413	0.412
Secondary_Metabolism	0.307	0.259	0.343	0.27	0.154	0.164
Transcriptional_regulation	0.256	0.278	0.233	0.226	0.15	0.143
Dormancy_and_Sporulation	0.219	0.176	0.145	0.143	0.129	0.131
Virulence,Disease_and_Defense	0.117	0.144	0.128	0.141	0.114	0.107
Photosynthesis	0.045	0.113	0.099	0.247	0.088	0.086
Phages,Prophages,Transposable_elements	0.007	0.014	0.008	0.008	0.008	0.006
Clustering-based_subsystems<br>Tartronate-semialdehyde_related_area_<br>(links_to_pyridoxine_and_aldorate_metabolism)	0.001	0.002	0.001	0.001	0.002	0.001
Arabinose_Sensor_and_transport_module	0	0.001	0.001	0	0	0
Cell_Division_and_Cell_Cycle,Bacterial_checkpoint_control	0	0.001	0.001	0	0	0
Plant_cell_walls_and_outer_surfaces	0	0	0.001	0	0	0

Table S11: Relative abundances for abundant (>1%), moderate (>0.1% and <1%) and rare (<0.1%) genera in the whole Archean Domes community and in the core community. Relative differences are shown for comparison.

Sample	Category	Relative abundances (%)		
		Whole community	Core community	Relative differences
Dry-2016	Abundant	67.63	67.63	0
	Moderate	19.65	18.87	3.98
	Rare	12.72	6.4	49.7
Wet-2016	Abundant	50.8	46.4	8.66
	Moderate	31.85	25.74	19.2
	Rare	17.35	6.52	62.44
Dry-2017	Abundant	46.84	45.61	2.62
	Moderate	35.53	28.91	18.62
	Rare	17.63	6.28	64.36
Wet-2018	Abundant	43.35	43.35	0
	Moderate	37.71	32.59	13.57
	Rare	18.94	5.5	70.96
Dry-2019	Abundant	52.44	45.8	12.65
	Moderate	36.01	23.88	33.69
	Rare	11.56	5.92	48.75
Wet-2019	Abundant	54.88	48.94	10.82
	Moderate	33.94	23.38	31.11
	Rare	11.18	5.2	53.53

Table S12: Alpha diversity indexes (Chao, Shannon, and Inverse Simpson) for each sample studied.

Sample/Index	Chao	Shannon	Inverse Simpson
Dry-2016	271.2500	2.792135	5.509622
Wet-2016	143.4000	2.642506	5.176839
Dry-2017	200.0833	2.592936	4.631164
Wet-2018	205.1154	2.716131	5.214591
Dry-2019	233.0000	3.128192	8.313074
Wet-2019	231.3750	3.077123	8.248053

Table S13: Network metrics for global network (phylum level) and core network (genus level). Hub taxa found by NetCoMi is also shown.

	Phylum-level Global network		Genus-level Core network	
Category/ parameter	Dry season	Rainy season	Dry season	Rainy season
Association threshold	0.5	0.5	0.5	0.5
Number of components	1.0	1.0	1.0	1.0
Clustering coefficient	0.75980	0.73311	0.11670	0.12789
Modularity	0.01279	0.06986	0.16715	0.21577
Positive edge percentage	49.01681	48.77283	41.94115	41.41117
Edge density	0.57415	0.56764	0.07316	0.07650
Natural connectivity	0.21904	0.20453	0.01452	0.01545
Vertex connectivity	3.0	1.0	6.0	7.0
Edge connectivity	3.0	1.0	6.0	7.0
Average dissimilarity	0.78332	0.78216	0.97908	0.97812
Average path length	0.82592	0.84478	1.45398	1.44128
Hub taxa	cand.div. WWE3<br>Hydrogenedentes<br>Omnitrophica<br>Pacebacteria<br>Planctomycetes<br>Sumerlaeota	Acidobacteria<br>Actinobacteria<br>Bacteria NA<br>Bipolaricaulota<br>Euryarchaeota<br>Planctomycetes	Chitinispirillum<br>Coleofasciculus<br>Desulfonatronovibrio<br>Desulfovermiculus<br>Halanaerobium<br>Halomonas<br>Halorhabdus<br>Halorubrum<br>Marinilabilia<br>Mariniphaga<br>Marinobacter<br>Spiribacter<br>Tangfeifania	Bradymonas<br>cand. Izimaplasma<br>Chitinispirillum<br>Coleofasciculus<br>Desulfohalobium<br>Desulfonatronovibrio<br>Desulfovermiculus<br>Hlanaerobium<br>Halomonas<br>Halorhabdus<br>Halorubrum<br>Halorussus<br>Rhodovulum
